# Effect of Nb^5+^ Doping on Structural, Electronic, and Antibacterial Properties of Hydroxyapatite

**DOI:** 10.1021/acs.inorgchem.6c01213

**Published:** 2026-05-25

**Authors:** Rafael Araújo, André Luiz Menezes de Oliveira, Brendan James Kennedy, Ary Silva Maia, Lúcio Castellano, Fábio Correia Sampaio, Santiago Medina-Carrasco, María del Mar Orta Cuevas, Maria Gardênnia Fonseca

**Affiliations:** † Núcleo de Pesquisa e Extensão LACOM, Dept. de Química, 28097 Universidade Federal da Paraíba, 58051-085 João Pessoa, PB, Brazil; ‡ Laboratório Institucional de Microscopia Eletrônica e Caracterização de Materiais (LIME), Dept. de Engenharia de Materiais, Universidade Federal do Rio Grande do Norte, 59078-970 Natal, RN, Brazil; § School of Chemistry, The University of Sydney, Sydney, NSW 2006, Australia; ∥ Escola Técnica de Saúde, Universidade Federal da Paraíba, 58051-900 João Pessoa, PB, Brazil; ⊥ Dept. de Clínica e Odontologia Social, Universidade Federal da Paraíba, 58051-900 João Pessoa, PB, Brazil; # CITIUS Laboratorio de Rayos X, 16778 Universidad de Sevilla, 4B 41012 Seville, Andalucía, Spain; ∇ Dept. de Química Análitica, Facultad de Farmacia, Universidad de Sevilla, E 41012 Seville, Andalucía, Spain

## Abstract

Herein, we address the limited understanding of how Nb^5+^ doping governs the defect chemistry and structural characteristics of hydroxyapatite (Hap) and demonstrate its use to tailor their functionalities. Niobium-doped Haps (HapNb*x*) were synthesized using the coprecipitation method, with Nb doping concentrations of *x* = 1, 3, 5, 7, and 10 mol %. The effect of dopant concentration on the structural and electronic properties of the samples was systematically investigated and correlated with their functionalities. Structural characterization and Rietveld refinements revealed that single-phase apatite-structured materials were obtained up to 7 mol % Nb, whereas phase segregation occurred at 10 mol %. Incorporation of Nb^5+^ into the Hap lattice was revealed by Raman, UV–vis, and XPS spectroscopic analyses and further confirmed by XANES spectroscopy at the P K edge and Nb L-edge. XPS analysis indicated the presence of this cationic species on the surface, and XANES probed the preferential coordination sites for Nb^5+^ cations. The antibacterial activity against *Staphylococcus aureus* and *Escherichia coli* was evaluated, and the sample containing 5 mol % Nb demonstrated a promising inhibitory effect. Our findings reveal that Nb^5+^ preferentially occupies the 7-coordinate Ca­(II) sites in the Hap lattice, thereby modifying the structural and electronic properties of HapNb*x* compounds, affecting their biocidal function.

## Graphical abstract

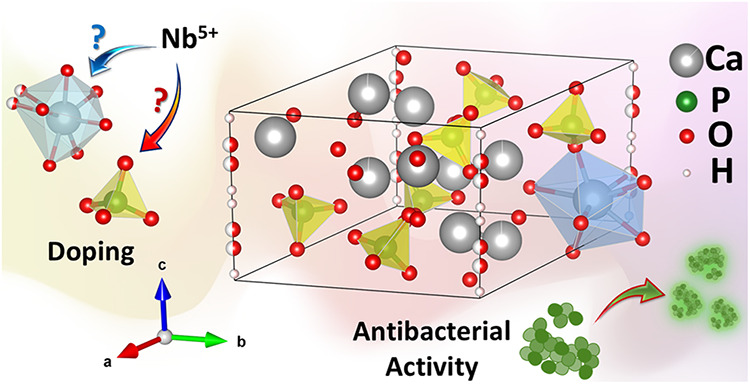

## 1 Introduction

Calcium phosphate-based bioceramics have gained considerable attention for orthopedic and dental applications,^1–3^ due to the chemical similarities between these minerals and bone tissue. Hydroxyapatite (Hap) with the chemical formula Ca_10_(PO_4_)_6_(OH)_2_ continues to be the main choice for biomedical applications, with clinical and therapeutic applications ranging from repair to replacement of damaged hard tissues.^4–7^

To expand the applications of Hap, numerous studies have modified its structure by introducing different types and amounts of metal cations into the lattice.^4,6–9^ One of the most recent strategies for modifying the Hap structure, and thereby conferring new and improved properties, is to incorporate Nb^5+^ cations into the material. Niobium has outstanding properties attractive to biomaterials, such as low density, nontoxicity, biocompatibility, and high chemical and mechanical resistance.^10–12^ Nb also exhibits bioactivity, and it can promote a high degree of osteoblast adhesion and proliferation.^13^ Thus, Nb is a promising candidate for application in antiallergic coatings for endoprostheses and bone repair.^14,15^

Nb-containing metallic alloys show useful properties for bone fracture treatment because they exhibit a low modulus of elasticity and excellent corrosion resistance.^16–18^ Additionally, Nb-modified vitreous materials exhibit improved mechanical properties and bioactivity^19^ and can enhance the differentiation and mineralization of osteogenic cells.^20,21^ Recent studies have highlighted the ability of Nb to enhance the bioactivity of Hap-Nb_2_O_5_ composites,^22,23^ originating from the formation of an extensive layer of surface precipitates when the Hap-Nb_2_O_5_ composites are exposed to a simulated body fluid.^24^

Several studies have demonstrated the potential bioactive effects of Nb^5+^ dopants in Hap-based materials. Tamai et al.^25^ showed that the incorporation of Nb into biphasic Hap/β-tricalcium phosphate (Hap/ β-TCP) ceramics enhanced the activity of human osteoblasts, resulting in increased calcified tissue. Thiyagarajan et al.^26^ used density functional theory (DFT) methods to understand the effect of Nb doping on biphasic Hap/β-TCP materials and correlated it with variations in their reactivity. Conversely, Capanema et al.^27^ prepared Hap bioceramics doped with 10 mol % Nb and showed that Nb doping improved their biocompatibility, resulting in an increased rate of osteoblast cell proliferation. These studies indicate that Nb-doped Hap can be utilized as a bone substitute. Recently, Korzeniewski and Witkowska^28^ synthesized calcium phosphate glass bioceramics doped with 10 and 20 mol % Nb and demonstrated that high Nb content altered the stability of the phosphate structure, leading to significant phase segregation. Nevertheless, there is a lack of reports describing a strategy for preparing single-phase Hap systems doped with Nb^5+^ that are necessary to clearly elucidate the impact of Nb^5+^ on structure, properties, and function.

Motivated by the importance of Hap and (Nb), we synthesized various Nb^5+^-doped Hap biomaterials via the coprecipitation method and evaluated their antibacterial properties. The incorporation of Nb at different concentrations (1, 3, 5, 7, and 10 mol %) and its effect on the average crystal structure, local chemical coordination, and electronic properties of Hap were studied and correlated with the antibacterial properties. This study expands the currently limited understanding of how Nb^5+^ doping governs the structural characteristics and defect chemistry of Hap. Moreover, by establishing the relationship between synthesis-structure-properties and biological performance, enables optimization of the material for biomedical applications. To the best of our knowledge, no such comprehensive investigation has been conducted on Nb^5+^-doped Haps. Therefore, this paper addresses an existing gap in the literature.

## 2 Experimental Section

### 2.1 Chemicals

Diammonium hydrogen phosphate ((NH_4_)_2_HPO_4_, Merck, 99%), calcium chloride (CaCl_2_·2H_2_O, Sigma-Aldrich, 93%), sodium hydroxide (NaOH, Vetec, 97%), and niobium ammonium oxalate ((NH_4_H_2_[NbO­(C_2_O_4_)_3_], CBMM, 99%) were used as reagents to prepare the materials, without further purification. Deionized water was used in all procedures.

### 2.2 Synthesis of Nb-Doped Hydroxyapatites (HapNb*x*)

Pure hydroxyapatite (Hap) particles were prepared according to a previously reported procedure.^29^ Initially, 250 mL of a 0.09 mol L^–1^ (NH_4_)_2_HPO_4_ solution and 250 mL of 0.15 mol L^–1^ CaCl_2_·2H_2_O solution were prepared and simultaneously added to the glass beaker at a flow rate of 1 mL min^–1^ under constant magnetic stirring at 1000 rpm. The amounts of Ca and phosphate ions used in the synthesis were 0.56 and 0.33 mol, respectively, corresponding to a stoichiometric molar ratio of 1.67. During the synthesis, the pH of the system was adjusted to 10 using a 0.15 mol L^–1^ aqueous NaOH solution, and a white solid was formed. The white precipitate was aged for 24 h at 30 °C under magnetic stirring at 200 rpm, filtered, and washed with deionized water until chloride ions were eliminated from the solution. The solid was oven-dried for 24 h and finally calcined in a muffle furnace at 600 °C for 6 h. The calcination was carried out in air atmosphere at a heating and cooling rate of 5 °C min^–1^.

The same procedure was used for the synthesis of Nb-doped Hap (HapNb*x*) samples, differing only in the addition of the Nb precursor to the ammonium phosphate solution, followed by the addition of the precursor salt solutions containing the Ca and (P + Nb). The amount of doped Nb^5+^ was 1, 3, 5, 7, and 10 mol % of the ion relative to the amount of Ca (in %) in the molecular formula of Hap. The samples were designated as HapNb*x* (where *x* = mol % Nb).

### 2.3 Characterizations

X-ray Powder diffraction (XRD) patterns were recorded using a Bruker D8 Discover A25 diffractometer equipped with a Lynxeye position-sensitive detector, operating with Cu Kα radiation. Measurements were carried out over the 2θ range 5–120° with a scan rate of 0.002° s^–1^. The structural and microstructural refinements of the solids were performed using TOPAS 6 software.

The Fourier transform infrared (FT-IR) spectra were recorded on a Shimadzu Prestige–21 IR spectrophotometer in transmittance mode using KBr pellets over the wavenumber range of 400–4000 cm^–1^ at a resolution of 4 cm^–1^. For each spectrum, 30 consecutive scans were performed.

Raman spectra were acquired with a Renishaw inVia Micro-Raman spectrometer using a 514 nm diode laser and a 2400 l/mm grating.

X-ray fluorescence (XRF) results were obtained with a Shimadzu spectrometer (model XRF-1800) using Cu Kα radiation at 2 kVA, 30 kV, and 30 mA.

Field-emission scanning electron microscopy (FE-SEM) images of the samples were obtained using a Tescan MIRA3 microscope, while elemental mapping was performed on the same microscope coupled with an Oxford X-ACT IE150 energy-dispersive spectrometer (EDS).

UV–vis absorption spectra (UV–vis) were obtained on a Shimadzu UV-2550 spectrophotometer with a spherical integration accessory. The spectra were acquired over 190–900 nm, and pure barium sulfate (BaSO_4_) powder was used as a background correction standard.

X-ray photoelectron spectroscopy (XPS) measurements were performed using a ScientaOmicron ESCA+ spectrometer with a high-performance hemispherical analyzer (EA 125) set at a pass energy of 30 eV, and monochromatic Al Kα radiation (*h*ν = 1486.6 eV) was used as an excitation source. The operating pressure in the ultrahigh vacuum (UHV) chamber during the analysis was about 2 × 10^–9^ mbar. Energy steps of 0.5 and 0.05 eV were used for the wide-scan and high-resolution spectra, respectively. A charge neutralizer was used during the measurements. The binding energy of the spectrum was corrected using the C 1s photoemission line set at 284.8 eV.

Phosphorus (P) K-edge and niobium (Nb) L-edge X-ray absorption near-edge structure (XANES) data were collected in fluorescence mode under high vacuum conditions at the Medium Energy X-ray Absorption Spectroscopy beamline (MEX-2) at the Australian Synchrotron. The samples were finely ground, then dusted onto double-sided conducting carbon tape and attached to stainless steel sample holders. The energy calibration was conducted concurrently with the total electron yield (TEY) signals measured from the reference foils positioned upstream in the beamline. The spectra were normalized using the Athena software.^30^

### 2.4 Antibacterial Activity

The antimicrobial activity of the samples was evaluated using the adapted broth microdilution method, following the methodological principles described by the Clinical and Laboratory Standards Institute (CLSI)^31^ and Rodríguez-Melcón et al.^32^ The essays were performed against microorganisms from the American Type Culture Collection (ATCC), Gram-positive *Staphylococcus aureus* (ATCC15656) and Gram-negative *Escherichia coli* (ATCC5922) bacterial strains, all provided by the Oswaldo Cruz Foundation (FIOCRUZ, Brazil).

Initially, the stock culture strain, stored in a freezer, was removed and allowed to reach room temperature (RT) in a laminar flow hood. Then 600 μL of the inoculum was transferred to a sterile test tube containing 7 mL of brain heart infusion (BHI) medium. The tube was then incubated at ±37 °C for 24 h, after which it was centrifuged for 10 min to remove the supernatant. The sample was resuspended in sterile saline solution (0.9% NaCl) and adjusted using a microprocessor-controlled digital Bel Photonics UV-M51 UV–vis spectrophotometer. The absorbance was monitored at 600 nm until a standardized bacterial suspension corresponding to 0.5 on the McFarland scale was obtained, corresponding to 10^8^ colony-forming units per mL (CFU mL^–1^). This was then diluted in sterile BHI medium to give a final concentration of approximately 5 × 10^5^ CFU mL^–1^. This value is widely used in broth microdilution antimicrobial susceptibility assays.^31,33^

Antibacterial activity assays were performed in 24-well microplates with a final volume of 1.0 mL per well. Each well received 900 μL of BHI medium containing the samples at final concentrations of 25 or 50 μg mL^–1^, followed by the addition of 100 μL of standardized bacterial inoculum. A negative growth control consisting of BHI medium with a bacterial inoculum, without an antimicrobial agent, was prepared. The microplates were incubated at 37 ± 2 °C for 24 h under aerobic conditions. All experiments were performed in triplicate.

The bacterial viability and antibacterial activity of the samples were determined by counting CFUs. After the incubation period, 500 μL of the contents of each well were transferred to tubes containing 4.5 mL of sterile saline solution (0.9% NaCl), corresponding to an initial dilution of 10^–1^. From this suspension, successive serial dilutions were prepared up to 10^–6^. The colony count was selected to obtain plates within the ideal counting range (30–300 colonies), as recommended by CLSI.^31^ Aliquots of 10 μL of each dilution were seeded on BHI agar and incubated at 37 ± 2 °C for 18–24 h. After incubation, visible colonies were counted, and the results were expressed as CFU/mL according to the eq 1

1 $$\mathrm{CFU}/\mathrm{mL}=\frac{\mathrm{NC}\times \mathrm{DF}}{0.01}$$

where NC is the number of colonies and DF is the dilution factor. The values obtained were subsequently converted to logarithmic units (CFU/mL) to reduce experimental variability and facilitate comparison between treatments, in line with the approach adopted in similar studies.^32^

## 3 Results and Discussion

### 3.1 Structural Analysis by XRD and Rietveld refinements

According to XRD patterns depicted in Figure 1, pure Hap and Nb-doped Hap (HapNb*x*) samples exhibited diffraction peaks characteristic of Hap. The diffraction peaks were indexed to a hexagonal *P*6_3_/*m* structure (ICDD 00–09–0432).

**Figure 1:**
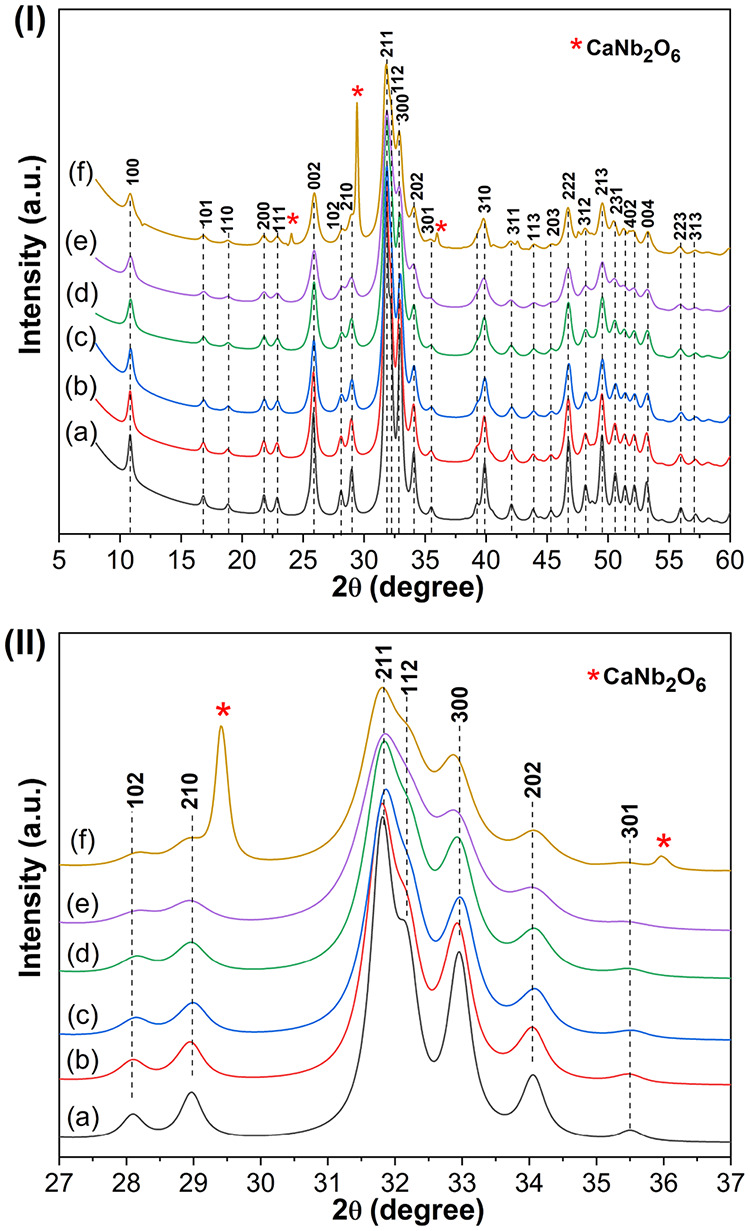
(I) XRD patterns and (II) magnified patterns over the 2θ range of 27–37° for (a) Hap, (b) HapNb1%, (c) HapNb3%, (d) HapNb5%, (e) HapNb7%, and (f) HapNb10% samples.

The analysis of the diffraction patterns revealed the crystallization of single-phase HapNb*x* solids for all samples up to *x* = 7 mol %. The materials were apparently stable, although they exhibited decreased crystallinity with increasing Nb content. The XRD pattern of the HapNb10% sample showed additional diffraction peaks corresponding to an undesired secondary phase, indicating that 10% Nb doping exceeded the limit for preserving the Hap structure. The XRD data are consistent with the literature for the same type of modification. For example, de Amorim et al.^34^ modified Hap with Nb, although they limited the dopant concentration to ∼ 1%, and observed the presence of calcium niobate (CaNb_2_O_6_) and niobium pentoxide (Nb_2_O_5_) by conventional XRD. Our observations agree with those of Korzeniewski and Witkowska^28^ who reported that significant phase segregation occurs upon doping calcium phosphate glass ceramics with high Nb concentrations (10 and 20 mol %).

To better understand the effect of Nb doping on the crystal structure of Hap, Rietveld structural refinements were performed for all the synthesized samples. The Rietveld profiles are shown in Figure 2. The selected results are listed in Tables 1 and S1–S2 of the Supporting Information file (SI).

**Figure 2:**
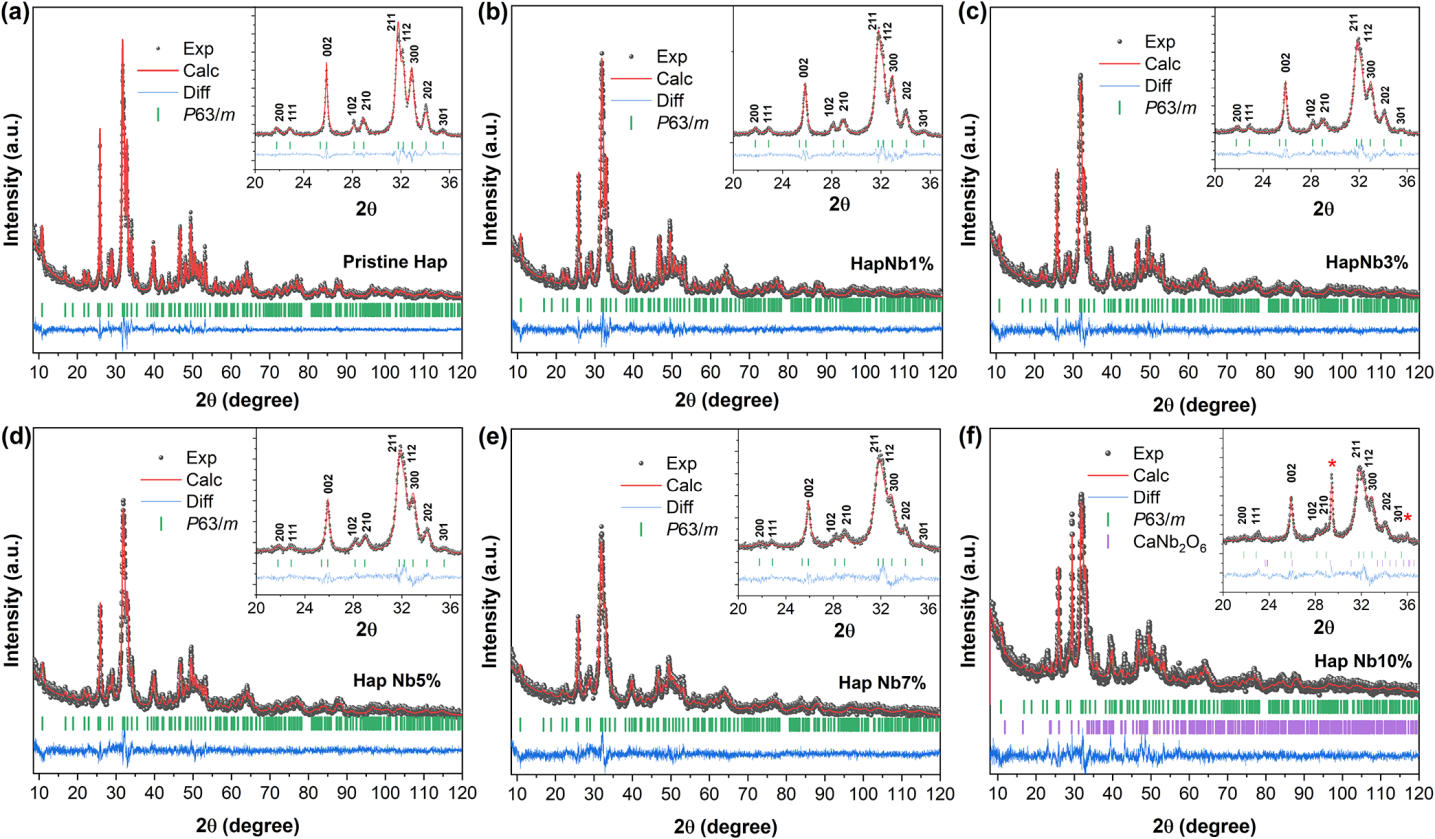
Rietveld XRD profiles of (a) pristine Hap, (b) HapNb1%, (c) HapNb3%, (d) HapNb5%, (e) HapNb7%, and (f) HapNb10% samples. The insets depict a zoom into the XRD region of 2 θ = 20–37°.

**Table 1:** Refined Lattice Parameters (*a* and *c*), Unit Cell Volume (*V*), and Measures of Fit for HapNb*x* (*x* = 0, 1, 3, 5, 7, and 10 mol % Nb) Samples in the Hydroxyapatite Structure^a^

	Hap	HapNb1%	HapNb3%	HapNb5%	HapNb7%	HapNb10%
Composition	HAp	Hap	HAp	HAp	HAp	HAp/CaNb_2_O_6_
Phase fraction (%)	100	100	100	100	100	98.78/1.22
Space group	*P*6_3_/*m*	*P*6_3_/*m*	*P*6_3_/*m*	*P*6_3_/*m*	*P*6_3_/*m*	*P*6_3_/*m*/*Pbcn*
*a* (Å)	9.4308(17)	9.4014(10)	9.3984(12)	9.4113(14)	9.416(2)	9.440(3)
*c* (Å)	6.8858(12)	6.8802(8)	6.8800(10)	6.8788(11)	6.8782(16)	6.885(2)
*V* (Å^3^)	530.4(2)	526.64(12)	526.29(16)	527.65(18)	528.1(3)	531.4(4)
GOF	1.32	1.17	1.32	1.20	1.19	1.32
*R*_wp_	12.80	11.14	11.09	10.75	10.05	11.08
*R*_Bragg_	3.352	2.752	2.744	2.830	2.603	2.856/6.511

a Further details are given in the SI.

The structural analysis shows that all samples, except HapNb10%, were single-phase and crystallized with a hexagonal structure in space group *P*6_3_/*m*. Analysis of the XRD pattern for HapNb10% revealed the existence of a secondary phase identified as CaNb_2_O_6_, indexed by ICDD 04–001–7554, space group *Pbcn*, as reported by Amorim et al.^34^ The structural refinement for HapNb10% indicated the presence of 1.22 wt % CaNb_2_O_6_ in the sample. The refined lattice parameters for CaNb_2_O_6_ were *a* = 15.023(8) Å, *b* = 5.178(3) Å, *c* = 5.147(3) Å, and *V* = 442.1(4) Å^3^. Notably, even at the highest dopant concentration, diffraction peaks indicative of crystalline Hap were observed, a phenomenon not reported in the earlier study by de Amorim et al.^34^

Lima et al.^7^ reported that the doping of Hap with Zn^2+^ cations at the Ca^2+^-sites led to the merging of the Bragg reflections associated with the (211) and (300) planes, indicating an increase in long-range disorder in the crystal structure. Similar behavior was observed in the present case. Additionally, the diffraction peaks shifted to higher angles with increasing Nb^5+^ content up to 3 mol % Nb, indicating a decrease in the lattice parameters and contraction of the unit cell volume. The refined values (Table 1) show that the lattice parameter *a* and unit cell volume *V* decreased up to 3 mol % Nb^5+^ and increased with further increase in dopant concentration. The *c*-lattice parameter decreased slightly for samples containing up to 7 mol % Nb^5+^. This trend in the lattice parameters is better shown in Figure 3, and suggests that the contraction of the crystal structure is favored when the Ca^2+^ cations of Hap are partially replaced by cations with a smaller ionic radius, as observed by Paduraru et al.^35^

**Figure 3:**
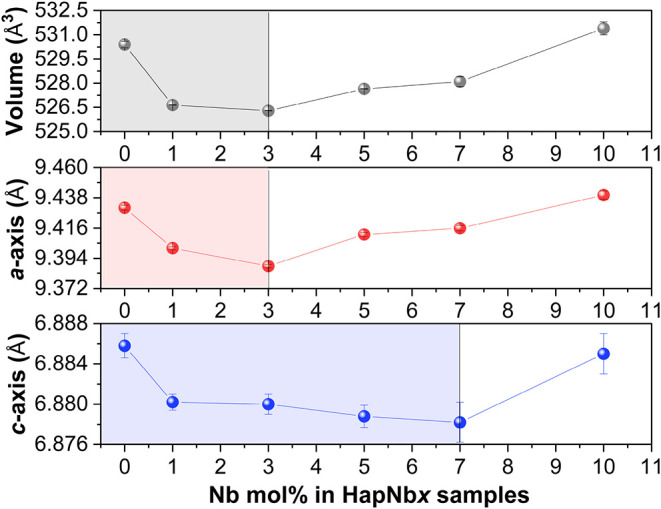
Lattice parameters and unit cell volume variation as a function of the Nb content in the Hap lattice.

The initial decrease in the lattice parameters, *a* and *c*, as well as in the unit cell volume *V* in the samples with up to 3 mol % Nb, can be attributed to the difference between the ionic radii of Ca^2+^ and Nb^5+^ cations. The replacement of Ca^2+^ by Nb^5+^ cations requires the formation of cationic vacancies. Therefore, the neighboring anions shift toward the cationic vacancies, resulting in a local contraction of the lattice around the defects. This may reduce the cationic coordination of oxygen within the tetrahedra (PO_4_^3–^) groups, thereby shortening the P–O bond and leading to an overall decrease in the unit cell parameters and volume, as observed for samples with up to 3 mol % Nb (Figure 3). The unexpected increase in the unit cell volume after 3 mol % Nb may be due to high structural disorder and high defect density induced by doping. This phenomenon may favor the formation of disordered phases that are undetected in the conventional XRD patterns.

Ca^2+^ occupies two distinct sites in the Hap structure: the nine-coordinate 4*f* Ca­(I) site and the seven-coordinate 6*h* Ca­(II) site. According to Shannon,^36^ the ionic radii of Ca (*r*_Ca^2+^_) in sites (I) and (II) are 1.18 and 1.06 Å, respectively. The ionic radius of the 7-coordinate Nb^5+^ (*r*_Nb^5+^_ = 0.69 Å) is significantly smaller than that of *r*_Ca^2+^_ in either site. Nb^5+^ is also known to be four-coordinate with an effective ionic radius of 0.48 Å, which is appreciably larger than the four-coordinate P^5+^ 0.17 Å. Given the size of the ions, it is likely that Nb will preferentially occupy the 7-coordinate Ca­(II) sites, although there is still no consensus on the preferred site for their occupation.

According to Capanema et al.,^27^ the substitution by Nb^5+^ occurs preferentially at the phosphate site, producing an increase in the lattice parameters and unit cell volume. However, this was not the case in the present study. Modifications with up to 1% dopant concentration have been reported to result in substitution at the Ca­(I) sites.^37^ However, increasing the dopant concentration increases the tendency for cation occupation at the Ca­(II) site.^38^ In contrast, other studies have suggested that the preferential position of cations is determined by their ionic radii and electronegativities. Veselinović et al.^39^ reported that the Ca­(II) sites tend to be preferentially occupied by cations with larger ionic radii or greater electronegativity than Ca^2+^, thereby facilitating stronger bonding to hydroxyl groups. Such behavior has been observed for cobalt-doped Hap, with a maximum of 11.7 at. % Co incorporated at the calcium positions.^39^

The methodology used in the present study to establish the Nb cation distribution by Rietveld refinement against the XRD data is as follows. First, the occupancies of the two Ca sites were allowed to vary while constraining the P and various oxygen sites to be fully occupied. For all the Nb-doped samples, the refined occupancy of the Ca­(I) site was less than one, and that of the Ca­(II) site was greater than one. This shows that there is less electron density at the Ca­(I) site and more at the Ca­(II) site than that those expected for unsubstituted Hap, indicating that Nb selectively occupies the Ca­(II) site and that the Ca­(I) site is not fully occupied. When the occupancy of the P site was allowed to vary, irrespective of the Nb content, the final refined occupancy was within one estimated standard deviation (esd) of full occupancy. This demonstrates that Nb did not partially replace P in the doped samples, validating the initial assumption that Nb would preferentially occupied the Ca sites. Partially replacing Ca^2+^ at the Ca­(II) site in Hap with x-equivalents of Nb^5+^ requires the formation of 1.5 × cation vacancies at the Ca­(I) site to retain charge neutrality. The results, presented in Tables S1–S2, are in reasonable, but not perfect, agreement with the nominal concentration of Nb.

As previously stated, the replacement of Ca^2+^ by a pentavalent Nb^5+^ cations in Ca_10–*x*_Nb_*x*_(PO_4_)_6_(OH)_2_ (HapNb*x*) introduces additional positive charges in the system, which are primarily compensated by the formation of calcium vacancies (V_Ca_^″^), although alternative charge compensation mechanisms may occur in the Hap structure.^40^ The formation of possible defects is represented by eqs 2–4, which are expressed in Kröger-Vink’s notation.

2 $${\mathrm{Nb}}_{2}O_{5}\overset{\mathrm{HapNb}x}{\to }2{\mathrm{Nb}}_{\mathrm{Ca}}^{...}+3V_{\mathrm{Ca}}^{\prime \prime }+5O_{O}^{x}$$

As the concentration of Nb^5+^ dopant incorporated into the Hap structure increases, an increasing number of V_Ca_^″^ defects is required to maintain electroneutrality. Consequently, additional defect states emerge, including the clustering of defects associated with anionic vacancies. These are hydroxyl vacancies V_OH_^•^ (eq 3) or phosphate vacancies V_PO_4__^···^ (eq 4).

In addition to balancing the lattice charge, these defects induce cooperative lattice distortions, as discussed below.

3 $${\mathrm{Nb}}_{2}O_{5}\overset{\mathrm{HapNb}x}{\to }2{Nb}_{Ca}^{...}+4V_{Ca}^{\prime \prime }+2V_{OH}^{\cdot }+5O_{O}^{x}$$

4 $${\mathrm{Nb}}_{2}O_{5}\overset{\mathrm{HapNb}x}{\to }2{\mathrm{Nb}}_{\mathrm{Ca}}^{...}+6V_{\mathrm{Ca}}^{\prime \prime }+2V_{{\mathrm{PO}}_{4}}^{...}+5O_{O}^{x}$$

Evidently, an increase in the doping level invariably results in the formation of elevated cationic vacancies and the clustering of anionic defects. This results in a nonuniform charge distribution within the lattice, which is a significant factor in the structural behavior of samples with high defect densities.

The increased charge-compensating defects reduce the electrostatic energy, with the neighboring cations shifting toward cationic vacancies. This, in turn, causes cooperative distortions and reorientations of the structural units of the lattice. Further doping and increased defect concentrations promote long-range defect–defect interactions. Consequently, the lattice undergoes cooperative relaxation and expansion upon further doping, as shown in Figure 3. This phenomenon can result in variations in bond lengths and bond angles (Table S3), symmetry lowering, and phase transformations. It can be concluded that the balance between electrostatic forces and charge compensation is ultimately responsible for determining the limits of defect incorporation, local structural disorder, and the phase stabilization, as evidenced in the case of HApNb10%. This finding is consistent with the study reported by Korzeniewski and Witkowska,^28^ which demonstrated that elevated dopant levels (10 and 20 mol % Nb) alter the stability of the phosphate structure. This was attributed to the replacement of the PO_4_^3–^ units, leading to substantial phase segregation.

These analyses reveal that the incorporation of Nb^5+^ ions into the Ca^2+^ sites can introduce various defects in the lattice, including V_Ca_^″^, V_OH_^•^, and V_PO_4__^···^. The resulting defect complexes play a crucial role in controlling structural stability, phase evolution, and functional properties.

The preferential occupancy of Nb at the Ca­(II) sites is attributed to a combination of size and the greater electronegativity of Nb than that of Ca, which favors covalent interactions with the hydroxyl groups in the Hap structure,^41^ as was also observed by Ignjatović et al.^42^ for rare earth-doped Hap. It should be noted that in the Hap structure, the hydroxyl groups surround the Ca­(II) sites.

The incorporation of Nb cations into the Hap structure increased the long-range structural disorder, as evidenced by the full-width-at-half-maximum (FWHM) values of the reflections along the (002), (211), (300), and (202) planes, as summarized in Table S4. The size of the crystallite domain decreased significantly by up to 7 mol % and then increased at 10 mol % Nb, possibly due to phase segregation and CaNb_2_O_6_ formation. The decrease in crystallite size upon Nb doping may be related to the increase in nucleation sites induced by Nb^5+^ cations in solution, which could inhibit crystallite growth and thereby reduce the crystallinity of the material. Similar behavior was been observed by de Lima et al.^7^ and Noori et al.^6^ for other doped Hap materials.

### 3.2 Chemical Analysis by XRF

The composition of the samples was investigated by X-ray fluorescence (XRF). XRF analysis suggested that the chemical composition of Ca and P in the samples was the same, in addition to the presence of Nb in the doped samples (Table 2). Based on these results, it could be concluded that the Ca/P ratio in the Hap studied here was 1.66. This value is very close to that of the stoichiometric Hap (Ca/P = 1.67) reported in literature, which indicates the efficiency of the method chosen to synthesize the materials.

**Table 2:** Chemical Composition Analysis by XRF Measurements for Pristine Hap and HapNb*x* Samples^a^

samples	CaO (wt %)	P_2_O_5_ (wt %)	Nb_2_O_5_ (wt %)	Ca (mol)	P (mol)	Nb (mol)	(Ca + Nb)/P
Hap	56.72	43.28	–	1.01	0.61	–	1.66
HapNb1%	54.65	42.53	2.81	0.97	0.60	0.021	1.67
HapNb3%	51.69	41.36	6.89	0.92	0.58	0.052	1.68
HapNb5%	50.55	41.48	6.79	0.90	0.60	0.051	1.59
HapNb7%	47.76	40.98	10.99	0.85	0.58	0.083	1.61
HapNb10%	44.09	37.94	17.73	0.79	0.53	0.133	1.74

a The main components of the modified solids, Ca, P, and Nb, are given.

The Ca and P concentrations in the samples decreased gradually with increasing Nb doping (Table 2), in agreement with the results obtained using other characterization techniques. Consequently, the (Ca + Nb)/P ratios estimated for the Nb-doped Hap samples were close to the stoichiometric ratio of pure Hap, probably indicating that Nb^5+^ compensated for the Ca^2+^ deficiency by replacing it in the Hap structure. The (Ca + Nb)/P ratio decreased at 5 and 7 mol % Nb, which is consistent with the formation of cationic vacancies in the Ca­(I) site, as established by the Rietveld refinements.

Surprisingly, a decrease in Ca content is accompanied by a significant reduction in P content, which may be due to the charge compensation mechanism in the lattice resulting from Nb^5+^/Ca^2+^ substitution. The partial replacement of Ca^2+^ cations with pentavalent Nb^5+^ cations introduces an excess of extra positive charges, and the structure may compensate for this by creating cationic vacancies, especially in the phosphate PO_4_^3–^ units. Consequently, the apparent P content decreases because there are fewer phosphate groups per unit formula, as suggested by the XRF results. Furthermore, the decrease in P content in samples containing up to 7 mol % Nb may be due to some phosphate precursors remaining unreacted and being lost from the solution during the doping process, in which cations compete for binding sites. Additionally, we cannot rule out the presence of disordered or amorphous phosphate-related domains within the samples that are undetected by conventional X-ray powder diffraction. Increasing the Nb content to 10 mol % significantly increased the (Ca + Nb)/P ratio, which may be related to the formation of calcium niobate as a secondary phase, as determined by the XRD and Raman spectroscopy analyses for HapNb10%.

### 3.3 Morphology and Qualitative Microanalysis by FE-SEM/EDS

To investigate the particle morphology and elemental composition mapping in synthesized solids, pristine Hap and HapNb*x* samples modified with up to 7 mol % Nb^5+^ were characterized by FE-SEM/EDS (Figures 4a,b and S1–S4). FE-SEM images (Figure 4a) showed the presence of dense aggregates formed by fine nanoparticles with a slightly rounded shape and undefined edges, especially in Nb-containing samples. This is better illustrated for the sample HapNb1%, which exhibited nanoparticles with a size of approximately 62 nm (Figure S1b). Apparently, the pristine Hap sample forms a dense agglomeration of slightly elongated particles, as observed by Oliveira et al.^43^

**Figure 4:**
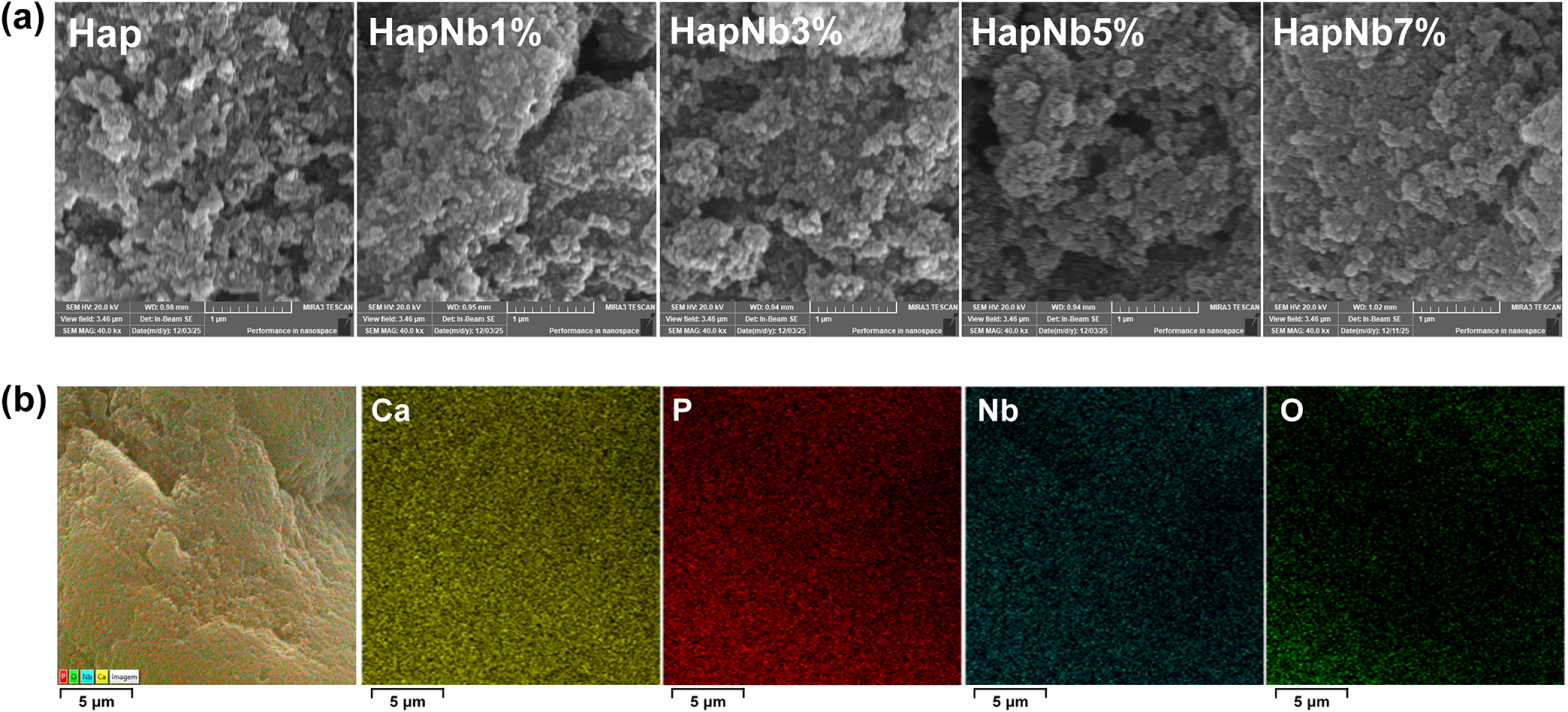
FE-SEM images for pristine Hap and HapNb*x* samples (a) and EDS elemental mapping (b) for HapNb5% sample.

The EDS elemental maps performed for the Nb-containing samples (Figures S1–S4), and highlighted here for HapNb5% (Figure 4b), confirmed the homogeneity of the constituent elements throughout the samples. Furthermore, no particles associated with the CaNb_2_O_6_ phase were observed, consistent with the Rietveld analyses.

### 3.4 Local Structural Order by FT-IR and Raman Spectroscopy

The FT-IR spectra (Figure 5) show characteristic IR bands for Hap below 1100 cm^–1^ for all the samples, as indicated by literature.^7,43–45^

**Figure 5:**
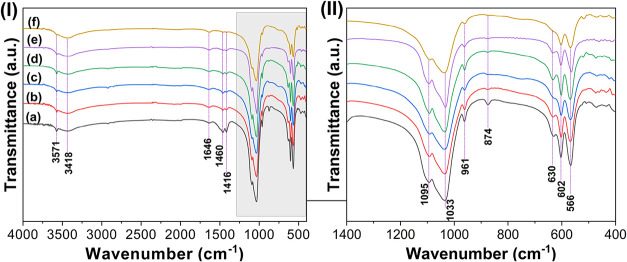
(I) FT-IR spectra and (II) magnified IR spectra over the wavenumber of 1400–400 cm^–1^ for Hap (a), HapNb1% (b), HapNb3% (c), HapNb5% (d), HapNb7% (e), and HapNb10% (f) samples.

The IR bands at 1095, 1033, and 961 cm^–1^ were attributed to the phosphate PO_4_^3–^ vibrational modes.^46^ Specifically, the bands at 1095 and 1033 cm^–1^ correspond to the asymmetric stretching of PO_4_^3–^, while that at 961 cm^–1^ is related to the symmetric stretching modes of the P–O bonds of the phosphate group. The bands at 602 and 566 cm^–1^ were assigned to the O–P–O deformation modes of the PO_4_^3–^ groups.^34,44^ With increasing Nb content, the intensities of the bands corresponding to the phosphate groups decreased, and the peaks broadened. This may be associated with the charge compensation processes because the charges and ionic radii of Ca^2+^ (*r*_Ca^2+^_ = 1.06 Å) and Nb^5+^ (*r*_Nb^5+^_ = 0.69 Å) are significantly different. Moreover, this type of substitution requires the formation of cationic vacancies, which can reduce the cationic coordination of an oxygen within the tetrahedra (PO_4_^3–^), thereby shortening the P–O bond. The shortening of the P–O bond can lead to a loss of symmetry and broadening of the absorption bands related to the PO_4_^3–^ groups, as indicated by the increased FWHM values of the main IR band at 1033 cm^–1^ (Table S5). This agrees with the results reported by Ignjatović et al.^42^ and Gomes et al.^47^

The increase in the FWHM values of the main IR band of the phosphate group with increasing Nb^5+^ content may indicate short-range disorder in the phosphate group.^42^ However, this modification was insufficient to destabilize the phosphate groups, indicating the structural stability of these materials, as observed by XRD. As reported by Korzeniewski and Witkowska,^28^ the incorporation of high concentrations of Nb (10 and 20 mol %) has a significant impact on the stability of the calcium phosphate; instability causes the formation of segregated phases of Nb_2_O_5_, Ca­(OH)_2_, and NH_4_CaPO_4_·H_2_O. Structural destabilization has been shown to be associated with an increase in defect concentration resulting from the replacement of PO_4_^3–^ sites by anionic Nb clusters.^28^

The band at 3571 cm^–1^ was attributed to the hydroxyl OH stretching, while the band at 630 cm^–1^ corresponds to the O–H deformation of the Hap structure. The absorption bands at 3448 and 1646 cm^–1^ were assigned to the stretching vibrations of the OH groups and the H–O–H deformation of physically adsorbed water molecules, respectively.^48,49^ A comparison between the spectra of the Hap and Nb^5+^-doped Hap samples revealed that the band attributed to structural hydroxyl underwent a significant decrease in intensity with increasing Nb content, possibly due to charge compensation. The OH- group may have been partially replaced by O^2–^ groups.

In addition to these bands, absorption bands related to the carbonate groups (CO_3_^2–^) were also observed at 1416, 1460, and 874 cm^–1^. These bands are commonly observed in Hap samples and can be associated with either surface carbonation due to the sample exposure to air postsynthesis or the formation of type B carbonated material,^50^ which can occur if the synthesis is conducted under basic media and under an uncontrolled atmosphere.^34,50^ The intensity of the bands related to the CO_3_^2–^ groups decreased with increasing Nb content, and the band disappeared completely for the solid with the highest concentration of Nb^5+^, as also observed by de Amorim et al.^34^ This may also be associated with charge compensation to stabilize the structure. Based on our analysis, it can be concluded that the Nb^5+^ cations are preferentially incorporated at the Ca sites, as indicated by the Rietveld refinements, and not at the P sites, as suggested in other studies.

The Nb^5+^/Ca^2+^ substitution may involve local structural distortions and cooperative effects in the crystal lattice due to the need for doping-induced point defects, primarily cationic vacancies, as described by Kröger-Vink’s notation and suggested by the Rietveld analysis. Nevertheless, our observed data agree with a previous DFT study that optimized the Hap structure doped with tri- and tetravalent cations.^51^ This DFT study revealed that modifications involving the P sites were unfavorable owing to strong P–O interactions, resulting in high energies and thus rendering the system unviable.

The short-range structural order in the HapNb*x* samples was also investigated using Raman spectroscopy, as shown in Figure 6. Active modes characteristic of the Hap structure were observed in all samples, with a decrease in the spectral resolution observed upon Nb^5+^-doping. The symmetric P–O stretching band of PO_4_^3–^ (ν_1_) in the Hap structure appeared at 961 cm^–1^.^52^ The bands at 429 and 446 cm^–1^ could be attributed to the ν_2_ O–P–O deformation modes,^53^ while the bands at 1049 and 1073 cm^–1^ corresponded to the ν_3_ asymmetric P–O stretching modes. The bands at 591 and 617 cm^–1^ were assigned to the ν_4_ O–P–O deformation modes.^54^

**Figure 6:**
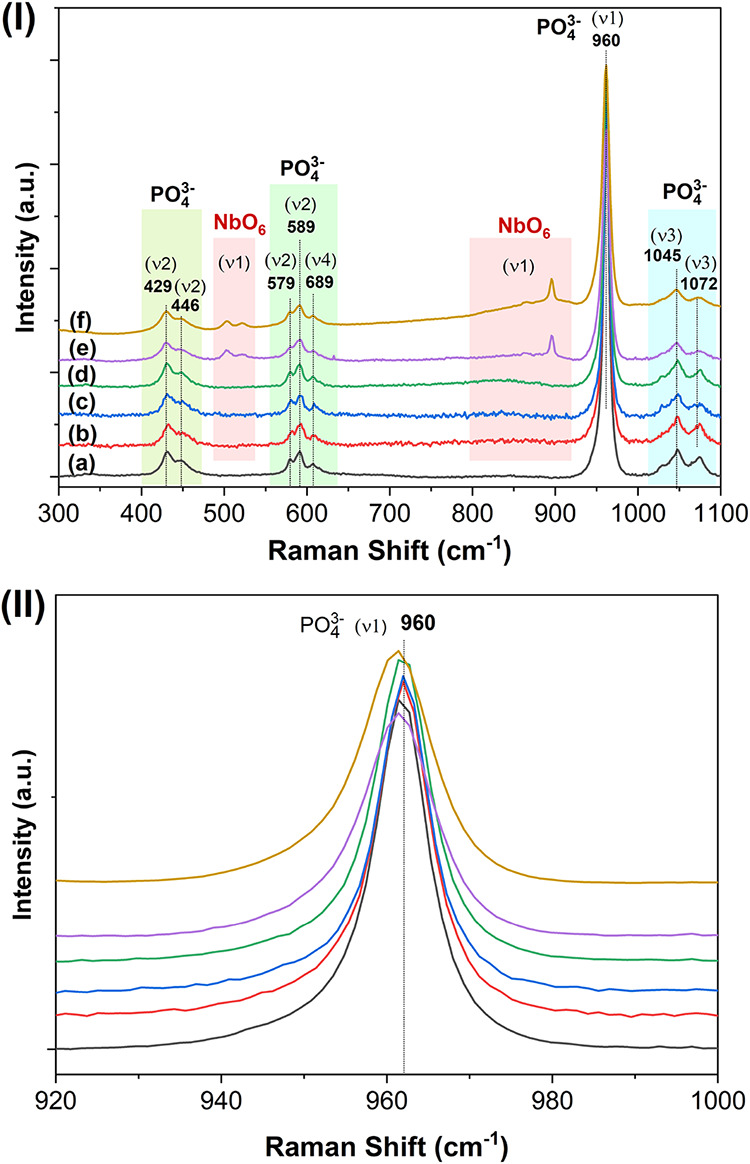
Raman spectra of the Hap (a), HapNb1% (b), HapNb3% (c), HapNb5% (d), HapNb7% (e), and HapNb10% (f) samples.

Changes in the Raman spectra were observed after the insertion of the Nb^5+^ cations into the Hap lattice, particularly for the solids prepared with 5, 7, and 10 mol % Nb. A broad feature appeared from 800 to 900 cm^–1^, which can be attributed to the Raman modes of the [NbO_6_]^7–^ octahedral units. The band associated with the NbO_6_ octahedra was observed by Chen et al.^55^ and Zhu et al.^56^ in niobate samples synthesized using a hydrothermal method. In the present case, replacing Ca with Nb may lower the coordination number of the Nb^5+^ species at the Ca­(II) site, owing to a decrease in the number of structural hydroxyl groups used as a charge-compensation mechanism. This is in accordance with the FT-IR data.

The estimated FWHM values of the band in Raman at 960 cm^–1^ (Table S6) increased as a function of the Nb concentration, which may indicate a loss of symmetry and an increase in the short-range structural disorder in the samples, in agreement with the Rietveld refinements, FT-IR analysis, and literature data.^7,47^ Furthermore, the formation of the secondary phase in the HapNb10% sample can increase the crystalline stress with the majority phase of Hap, leading to an increase in the structural disorder in the sample.

### 3.5 Electronic and Optical Properties by UV–Vis

The UV–vis absorption spectra of the samples are shown in Figure S5. The UV–vis spectrum of the pristine Hap sample shows a broad band centered at 227 nm, which is attributed to the O^2^ → Ca^2+^ charge transfer from both Ca­(I) and Ca­(II) sites. This spectral feature was also observed by Carniti et al.,^57^ who proposed the same structural modification of Hap with Nb cations by coprecipitation or by Nb deposition via impregnation on the presynthesized Hap sample. However, these authors did not conduct in-depth research to determine whether Nb species were incorporated into the apatite structure or chemisorbed on the surface as intended.

Unlike Carniti et al.,^57^ we found that the UV–vis spectral feature of the HapNb*x* samples was completely different from that observed for the undoped Hap. In our study, the intensity of the primary UV–vis absorption band, associated with O^2^ → Ca^2+^ charge transfer in Hap, decreases and is accompanied by a shift in the maximum band to a longer wavelength after Nb doping. This may be attributed to the Nb^5+^/Ca^2+^ replacement in the Hap lattice. Solids, especially those with the highest Nb content (HapNb10%) absorbed throughout the visible region, which can possibly be attributed to the O^2^ → Nb^5+^ ligand-to-metal charge transfer (LMCT) in the NbO_6_ octahedra, according to Scotti et al.^58^ and Wu et al.^59^ Additionally, no signals were observed at 230 nm in the spectra of the HapNb*x* samples, which are attributed to the charge transfer from O^2–^ to Nb^5+^ in tetrahedral coordination, as suggested by Wu et al.^59^ The absence of this signal may indicate that Nb does not adopt tetrahedral coordination when incorporated into the Hap structure; instead, it has a higher coordination number, as indicated by the Rietveld refinements, reinforcing the hypothesis that the substitution occurs at the Ca­(II) sites.

### 3.6 Surface Chemical Properties by XPS

The chemical nature and surface elemental composition of the HapNb*x* solids were investigated using XPS analysis. This analysis revealed the presence of Ca, P, and O in Hap, in addition to the presence of Nb in all the Hap samples modified with Nb (Figure 7). The presence of Nb^5+^ is consistent with Rietveld refinements, EDS, XRF, and Raman results. A more in-depth analysis was conducted by deconvolution of the high-resolution XPS spectra, as shown in Figure 8 for HapNb7% and illustrated for the other samples in Figures S6–S9. The estimated binding energies (BEs) for the samples are listed in Table S7.

**Figure 7:**
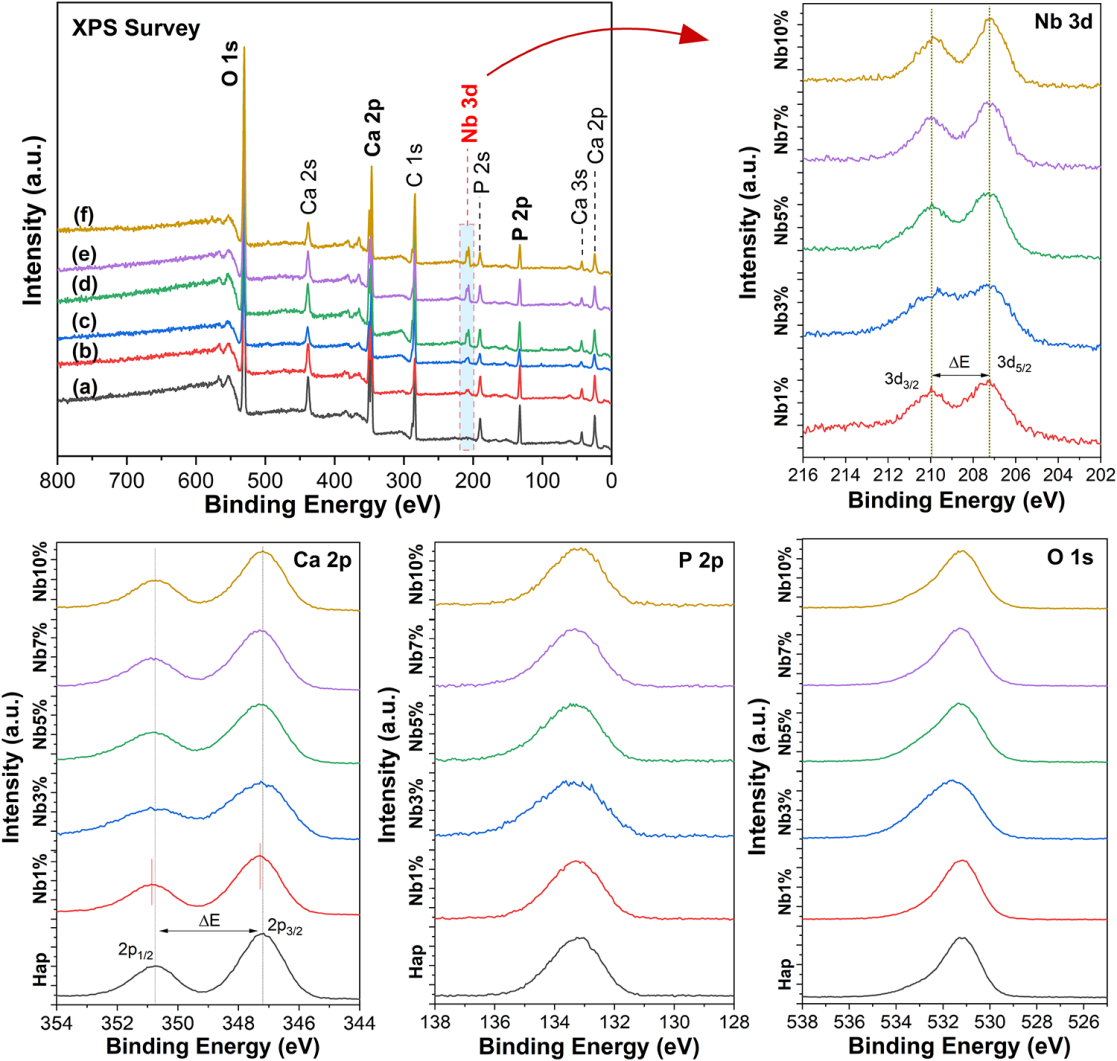
Overall XPS survey spectra and high resolution XPS spectra in Nb 3d, Ca 2p, P 2p, and O 1s emission lines of Hap (a), HapNb1% (b), HapNb3% (c), HapNb5% (d), HapNb7% (e), and HapNb10% (f) samples.

**Figure 8:**
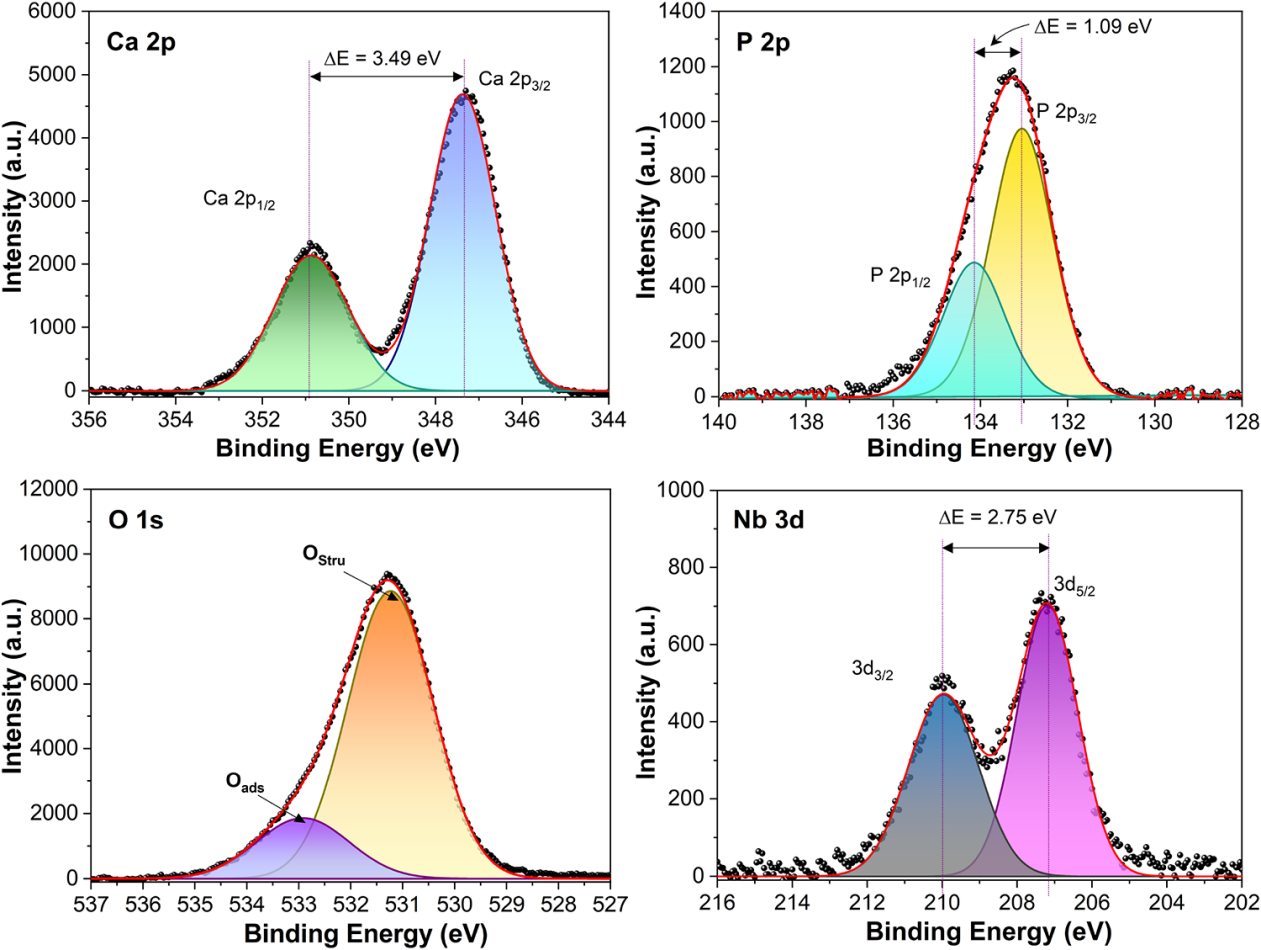
High-resolution XPS spectra deconvolution in Ca 2p, P 2p, O 1s, and Nd 3d emission lines for the HapNb7% sample.

Based on previous reports, the Ca 2p XPS spectra of the samples exhibit two well-defined peaks corresponding to the Ca 2p_3/2_ and Ca 2p_1/2_ levels.^7,43,44^ These peaks can be deconvoluted into two signals, with BEs varying according to the composition (Table S7). The doublet observed for the Ca 2p line in the pristine Hap sample has the binding energies of 350.77 eV, assigned to the Ca 2p_3/2_ line and 347.25 eV attributed to the Ca 2p_1/2_ level, yielding a spin–orbit energy (Δ*E*) of 3.55 eV, which is in agreement with those reported in the literature.^60,61^ After Nb^5+^ doping, broader and less intense peaks were observed in the Ca 2p spectra, particularly for the HapNb3% sample, as highlighted in Figure S6. Additionally, the peaks were slightly shifted to higher BEs. This behavior probably indicates Nb/Ca substitution in the Hap structure.

The high-resolution P 2p spectra exhibited a broad asymmetric peak owing to the overlap of the P 2p_3/2_ and P 2p_1/2_ lines.^57^ This asymmetric peak was also observed by de Lima et al.^7^ and Oliveira et al.^44^ for Zn-doped Hap. According to Bharath et al.^60^ and Lowry et al.,^61^ such an asymmetric profile is characteristic of phosphate-based compounds. The peaks related to the P 2p_3/2_ and P 2p_1/2_ states in undoped Hap were observed at 133.04 and 134.04 eV, respectively, with a spin–orbit coupling splitting (Δ*E*) of 0.99 eV. The broadening of the P 2p spectra was observed for the Nb-doped samples, probably indicating differences in the local environment of the phosphate groups on the Hap surface, as suggested by Bharath et al.^60^ This may be associated with the presence of PO_4_^3–^ vacancies induced by Nb doping.

The O 1s XPS spectra were also asymmetric, indicating overlapping signals that could be decomposed into two distinct peaks. The former was observed at 531.10 eV, which is attributed to structural oxygen (O^stru^) from the O–P bond of the phosphate tetrahedra and OH groups of the Hap structure. The latter appeared at 532.13 eV, corresponding to chemically adsorbed oxygen species (O^ads^) on the surface of the samples.^62^

For the HapNb*x* samples, two well-defined peaks are observed in the Nb 3d XPS spectra, which are absent in the spectrum of pure Hap. The absolute intensities of these increase with increasing Nb content, reflecting the higher proportion of Nb in the samples. These peaks correspond to the Nb 3d orbitals and can be deconvoluted into a simple 3d_3/2_ and 3d_5/2_ doublet at 207.30 and 208.49 eV, respectively. These BEs are characteristic of the Nb^5+^ species,^63–66^ further confirming that the Nb^5+^ cations compose the surface structure of all the synthesized samples.

### 3.7 Local Structure by P K-edge and Nb L-Edge XANES

Due to element-specificity and selectivity, as well as the sensitivity to local coordination and oxidation states of the selected elements in the structure, XANES spectroscopy was performed on the samples (Figures 9 and 10 and S10). This analysis allowed us to further probe the local Nb^5+^/Ca^2+^ or Nb^5+^/P^5+^ site-substitution in the Hap lattice. Figures 9 and 10 show the P K-edge and Nb L-edge XANES spectra for the samples, respectively. For comparison, the Nb L-edge XANES spectra of standard references were also collected (Figure S11).

**Figure 9:**
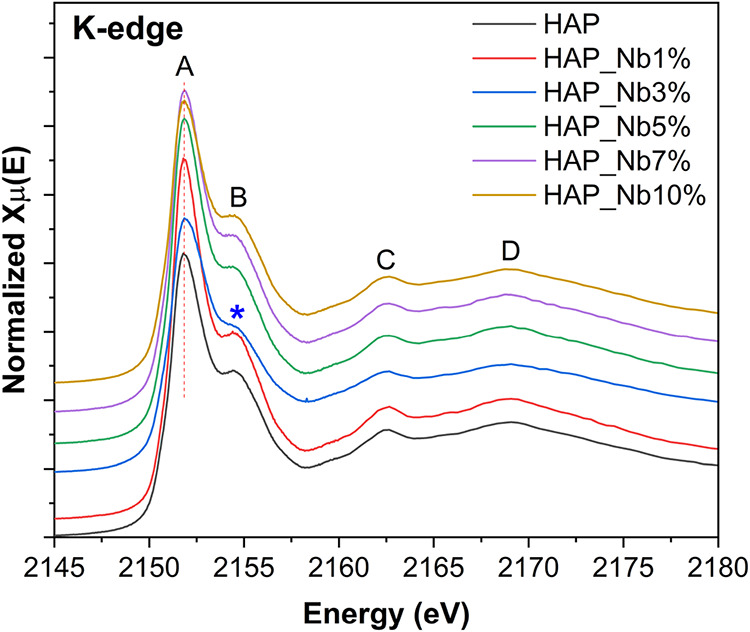
Phosphorus K-edge XANES spectra of the studied Hap and HapNb*x* samples.

**Figure 10:**
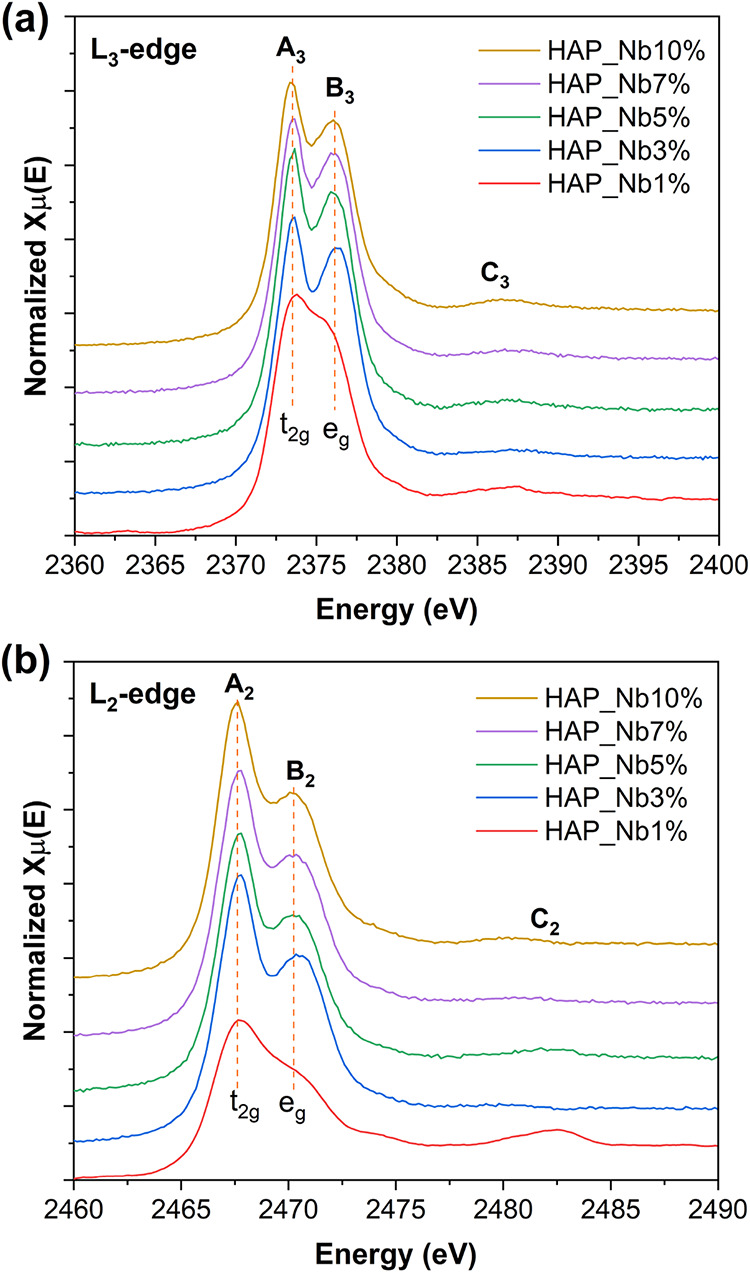
Niobium L_3_ (a) and L_2_ (b) edges XANES spectra of the studied HapNb*x* samples.

As shown in Figure 7, the P K-edge XANES spectra exhibit the typical features of a 4-fold coordination environment of P^5+^ cations, namely a tetrahedral geometry assigned to the phosphate (PO_4_^3–^) units.^67^ The overall spectral features are largely the same across the samples (Figure S10), typical of P K-edge XANES in the apatite structure and consistent with the literature.^68^

Two well-resolved bands were observed in the P K-edge XANES spectra: a highly intense white line at 2151.8 eV (Peak A), which corresponds to the dipole-allowed 1s → 3p transition due to the hybridization of P 3sp^3^ and O 2p orbitals in tetrahedral symmetry,^67^ and a distinct white-line shoulder at 2154.4 eV (Peak B), which is due to Ca-bound P in Hap.^69^ Notably, the intensity of Peak B in the spectrum of sample HapNb3% decreased (marked with *). This suggests that the local bonding environment around P is slightly changed, suggesting a minor distortion of the PO_4_^3–^ tetrahedra in this sample, induced by doping. This modifies the local ligand field, slightly altering the electronic density around the P cations. Postedge peaks at 2162.5 eV (Peak C) and 2168.9 eV (Peak D) were observed in the spectra. These are characteristic of the Hap structure^70^ and correspond to Ca–P and oxygen oscillation in the lattice.^69^

Apart from these features, no appreciable shift in the white lines was observed across the samples. This indicates that the fundamental phosphate (PO_4_^3–^) environment is preserved upon Nb incorporation, maintaining similar composition in terms of P species,^67,70^ regardless of the doping concentration.

The Nb L-edge XANES spectra collected on Nd-doped Hap samples are shown in Figure 10. The L_3,2_-edges are associated with the 2p_3/2_ → 4d transitions (Figure 10a), and 2p_1/2_ → 4d electronic transitions (Figure 10b), respectively. The L_3_- and L_2_-edges spectra are both characterized by the ligand field splitting of the empty Nb 4d orbitals into t_2g_ and e_g_ bands (using the octahedral labels for convenience). Similar features were observed in all samples, and the absorption edge positions were consistent with Nb^5+^ as indicated by comparison with reference materials analyzed in the present work (Figure S11), as well as with other standard references.^71^ Pre-edge and absorption edge variations and positions are therefore consistent with Nb^5+^ in octahedral coordination.

The XANES spectra show three peaks marked as *A*_3_ at 2373.4 eV and *B*_3_ at 2376.1 eV at the L_3_-edge (Figure 10a), and as *A*_2_ at 2467.6 eV and *B*_2_ at 2470.4 eV at the L_2_-edge (Figure 10b), and the energy splitting of the L_3_ and L_2_ edges is approximately 94.2 eV, consistent with the value reported in the literature.^71^ The separation of peaks *A*_3_ and *B*_3_ in the L_3_ XANES spectra is approximately 2.7–2.8 eV, which is significantly smaller than the 3.7 eV separation observed for NaNbO_3_ that contains Nb^5+^ in an almost regular octahedral geometry (Figure S11). The smaller ligand field splitting observed for the Nb-doped Hap samples indicates that Nb occupies the Ca­(II) site with longer Nb–O bond distances. Notably, no significant edge shift was observed, confirming that Nb maintains a 5+ oxidation state regardless of the amount of doping. The coordination environment of the Nb^5+^ cations in the samples can then be confirmed by the difference in the absorption intensities of the A and B peaks. This conclusion corroborates the XPS analysis, which indicated the presence of peaks in the Nb 3d XPS spectra, whose binding energies (BEs) were found to be associated with Nb^5+^.^72^ Additionally, the absence of additional peaks associated with Nb in lower oxidation states confirms the only presence of Nb^5+^ cations in the synthesized HapNb samples.

The higher intensity of peak A compared to peak B suggests that the Nb cations are in a distorted geometry, wherein the three t_2g_-derived orbitals are lower in energy than the two *e*_*g*_-derived orbitals.^59,71,73^ This is consistent with the NaNbO_3_ reference, which contains Nb^5+^ in octahedral geometry, as opposed to the spectral features observed for the YNbO_4_ and LuNbO_4_ references with Nb^5+^ atoms in a tetrahedral environment (Figure S11), as reported in the literature.^73^ In addition to these features, postedge peaks marked with *C*_3_ and *C*_2_ are observed in the spectra and are associated with 2p_3/2_ → 5s and 2p_1/2_ → 5s transitions, respectively.^59^

When the Nb L-edge XANES spectra of the HapNb*x* samples were compared, slight variations in the spectral broadening and peak intensities were observed, particularly at the *B*_3_ and *B*_2_ peaks associated with the e_g_ orbitals with increasing the Nb-content. Specifically, the features of the HapNb1% sample are not well-resolved, which may be due to the low Nb content in this sample and the different interaction of Nb with the surrounding oxygen. As the Nb content increases, the spectra become better resolved, with no significant white-line variations. However, minor spectral broadening and postedge oscillations were detected. In particular, a slight peak splitting of the corresponding t_2g_ and e_g_ states and the spectral broadening observed at both the L_3_ and L_2_ edges may indicate subtle modifications to the Nb–O bond lengths, leading to the distortion of the Nb octahedral site in the crystal lattice. This phenomenon has been reported by Bollaert et al.^71^ and Wang et al.^74^ for different compounds containing niobium. Changes in the local Nb environment corroborate with the structural adjustments in the lattice parameters and in the bond distances analysis (Table S3) estimated by the Rietveld refinements of the XRD patterns, as well as broadening observed in the Nb 3d XPS spectra.

Therefore, based on the P K-edge and Nb L-edge XANES results, the incorporation of Nb^5+^ cations into Hap does not disrupt the fundamental phosphate tetrahedral units, but may induce local distortions that also slightly alter the P–O electronic structure. However, the substitution of Ca^2+^ at the seven-coordinate 6*h* Ca­(II) sites lowers the coordination number of Nb, which adopts a 6-fold coordination in an octahedral site, as inferred from the Rietveld refinements. This structural and spectroscopic evidence supports the successful Nb^5+^/Ca^2+^ substitution, ruling out the replacement of P^5+^ by Nb^5+^ at the tetrahedral site throughout the doping range. Nevertheless, it is important to note that, despite the structural distortions, the Nb^5+^ cations remain octahedrally coordinated in the lattice. An illustration of the Hap structure, highlighting the Ca­(I), Ca­(II), and P coordination sites, as well as the preferential site for Nb­(V) incorporation, is shown in Scheme 1.

**Scheme 1:**
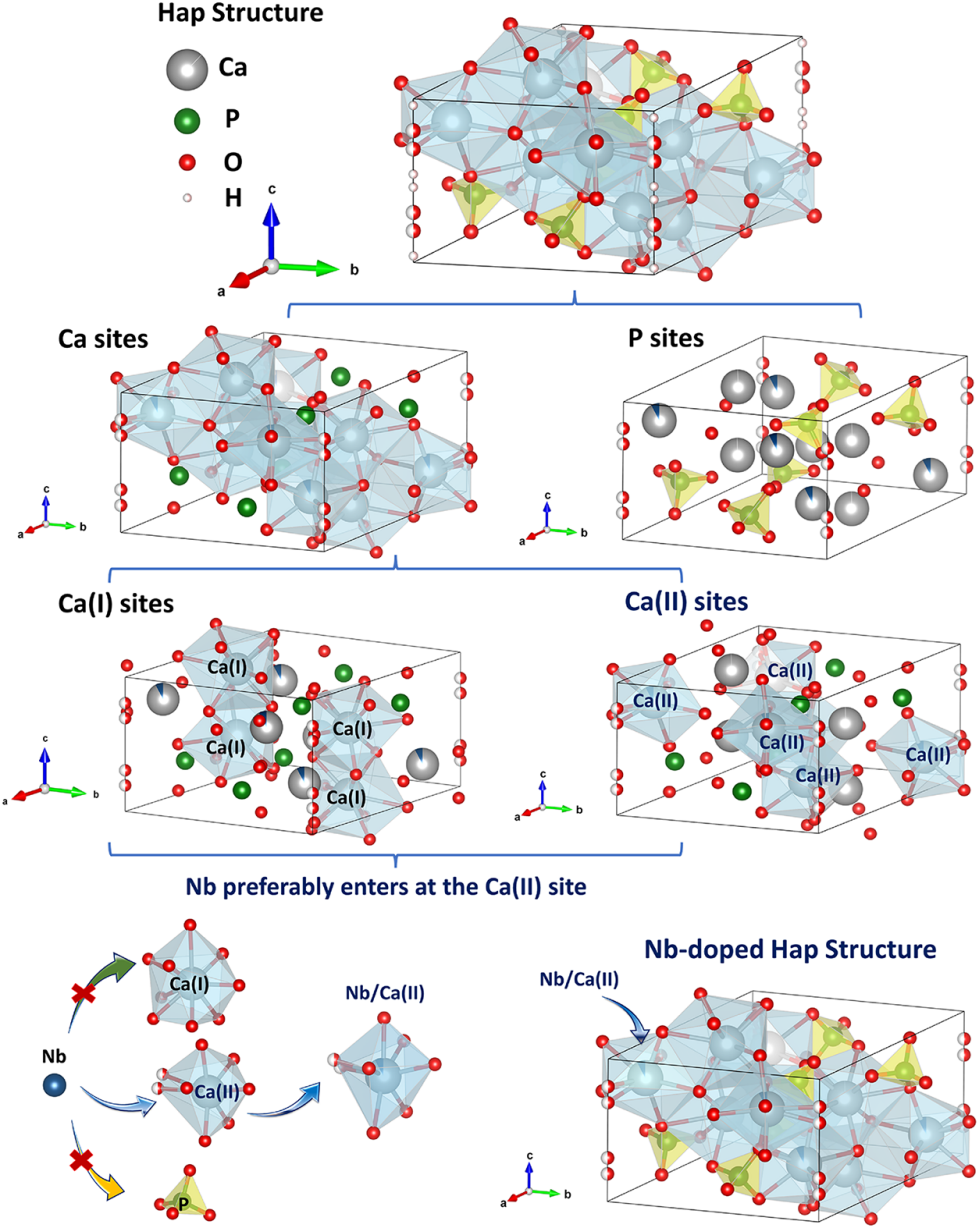
Illustration of the Hap Structure, Highlighting the Nine-Coordinate Ca­(I), Seven-Coordinate Ca­(II), and Four-Coordinate P Sites and the Preferential Substitution of Nb­(V) Cations into the Hap Structure to form the HapNb*x*-Type Compounds

### 3.8 Antibacterial Activity

The antibacterial effects of pure Hap and HapNb*x* samples were evaluated against *S. aureus* and *E. coli*, which were selected as the Gram-positive and Gram-negative bacteria, respectively. The cell wall architectures of these types of bacterial strains differ.^7,75^ The inhibitory effects of the samples are evident, as indicated by the results of the CFU count, expressed as logarithmic (CFU/mL) in Table 3. Different sample concentrations were evaluated and compared with a negative growth control.

**Table 3:** Effect of Increasing Percentage of Nb Dopant in Hap Samples on the Viability of *S. aureus* and *E. coli*, Expressed in log­(CFU/mL)

	*S. aureus*		*E. coli*	
sample	25 μg mL^–1^	50 μg mL^–1^	25 μg mL^–1^	50 μg mL^–1^
Hap	8.78	8.78	7.74	7.81
HapNb1%	8.60	8.48	7.56	7.23
HapNb3%	8.30	8.00	7.66	7.00
HapNb5%	8.00	<8.00	7.28	6.95
HapNb7%	8.00	8.00	7.59	7.15
Negative control	9.38	9.38	7.75	7.75

The results demonstrate that the antibacterial performance of the HapNb*x* samples depends on both the solid concentration and Nb doping level, with a clear concentration-dependent inhibition effect of Gram-positive *S. aureus* and Gram-negative *E. coli* bacteria. Conversely, the pristine Hap sample did not exhibit measurable antibacterial activity, especially against *E. coli* at any tested concentration, highlighting the critical role of Nb incorporation in activating the biocidal response.

The sample containing 5 mol % Nb exhibited the highest antibacterial activity against both bacterial strains. This enhanced performance is attributed to an optimal dopant concentration that promotes a favorable balance between structural and electronic properties, as indicated by the Rietveld refinements, XANES, and XPS analyses. At this moderate level of doping, the resulting structural order/disorder (associated with lattice distortions) and defect-state density (associated with cationic vacancies) do not compromise the integrity of the Hap framework. This optimal combination of structural disorder and defect density enhances charge transfer, both in the bulk and, most notably, at the sample surface. In turn, this yields the improved surface reactivity necessary to stimulate a controlled generation of reactive oxygen species (ROS).^76–78^ The controlled formation of ROS thereby enables efficient oxidative stress in bacterial strains.

In contrast, samples containing Nb at concentrations exceeding 5 mol % exhibited reduced antibacterial efficiency, despite the higher dopant level. This behavior is likely associated with excessive structural disorder, increased cationic vacancy concentration, and possible formation of disordered or amorphous domains that are undetected by conventional powder XRD analysis. Such effects reduce the charge distribution, leading to uncontrolled ROS production and impaired surface reactivity, thereby limiting antibacterial performance. The results indicate that the antibacterial activity is maximized within a narrow Nb doping window, in which defect generation and structural order/disorder are optimally balanced.

These findings are in agreement with previous reports on Nb-modified apatites. In particular, the positive effect of Nb doping on the antibacterial activity of Hap samples is consistent with the data reported by Saranya and Rani,^79^ who observed a significant antibacterial response for Nb-doped Hap solids at concentrations above 100 μg/mL against other Gram-positive bacteria, such as *Streptococcus oralis* and *Streptococcus pyogenes*, using the disk-diffusion method under direct contact conditions. This comparison confirms that Nb incorporation plays a decisive role in activating and enhancing the antimicrobial functionality of Hap. However, it still suggests that the antibacterial response depends not only on the material concentration but also on maintaining a controlled dopant level, because excessive Nb incorporation can lead to increased structural and electronic disorder and reduced functionality.

## 4 Conclusions

The present study addressed the limited understanding of how Nb doping governs defect chemistry and structural characteristics in Hap compounds to tailor their functional properties. The synthesis of Nb^5+^-doped Hap was achieved with the material exhibiting single-phase formation up to 7 mol % Nb and phase segregation at higher concentrations, thereby defining the solubility limit. XPS and XANES analyses confirmed that Nb is incorporated as Nb^5+^, preferentially substituting for Ca^2+^ at the Ca­(II) sites, with charge compensation occurring primarily through the formation of cationic vacancies. Increasing the Nb^5+^ dopant level induced structural and electronic disorder associated with anionic vacancies, which, in turn, directly affected phase stability and function. Enhanced antibacterial activity of the Nb-doped biomaterials against *S. aureus* and *E. coli* was achieved at intermediate doping levels, reflecting an optimal balance between defect density and structural integrity to stimulate ROS. The findings demonstrate that Nb^5+^-induced defect engineering provides an effective pathway for tuning the structure–property-biological performance relationships in Hap for biomedical applications.

## Supplementary Material



## Notes

Refined atomic positions; selected bond distances; selected bond angle variations; crystallite size; Full Width at Half Maximum (FWHM) for XRD patterns; FWHM values for IR; FWHM values for Raman; XPS Binding energies (BE) (Tables S1–S7); EDS spectra; EDS elemental mapping; UV–vis spectra; Deconvoluted XPS spectra; Normalized P K-edge XANES; and Nb L-edge XANES for standard references (Figures S1–S11) (PDF)

## References

[1] Guntu, R. K. Biobehavioral Structure, Elastic, Optical, and Thermoluminescence Properties of Al_ *x* _Ca_ *y*–*x* _Nd_01_P_70_ Bioglasses. In Bioresorbable Polymers and their Composites; Woodhead Publishing, 2024; pp 421–4442 10.1016/B978-0-443-18915-9.00006-9.

[2] Chand N. R. K., Sudhakar B., Ravikumar G., Gayathri V., Devika P., Vennela T., Rao G. S., Rao C. S. (2022). Influence of Multi
Valent States of Vanadium Ions in ZnO Doped Novel Calcium Fluoro Phosphate
Bio Glasses. J. Mech. Behav. Biomed. Mater..

[3] Kumar G. A., Rambabu Y., Guntu R. K., Sivaram K., Reddy M. S., Rao C. S., Venkatramu V., Kumar V. R., Iyengar N. C. S. N. (2021). Zr_
*x*
_Ca_30–*x*
_P_70_ Thermoluminescent
Bio Glass, Structure and Elasticity. J. Mech.
Behav. Biomed. Mater..

[4] Upadhyay A., Kayal D., Mandal S., Tripathi S., Parmar A. S., Kumar K., Mukherjee S. (2026). Fabrication
of 3D-Printed Adhesive
Microneedle Patch Loaded with Codoped Hydroxyapatite in Deep-Tissue
Infective Wound Healing. ACS Appl. Mater. Interfaces.

[5] Vieira E. G., Sousa R. B., da Silva M. P., Leal R. C., Vieira A. G., de Abreu W. C., Oliveira A. L., de Fonseca M. G., Carrasco S. M., Osajima J. A., Silva-Filho E. C. (2025). Development
of a Novel Ga-Containing Hydroxyapatite/Chlorhexidine Biomaterial
with Antibacterial Properties for Future Application in Bone Tissue
Engineering: An Experimental and Theoretical Study. Cerâmica.

[6] Noori A., Hoseinpour M., Kolivand S., Lotfibakhshaiesh N., Ebrahimi-Barough S., Ai J., Azami M. (2024). Exploring the Various
Effects of Cu Doping in Hydroxyapatite Nanoparticle. Sci. Rep..

[7] de
Lima C. O., de Oliveira A. L. M., Chantelle L., Silva Filho E. C., Jaber M., Fonseca M. G. (2021). Zn-Doped Mesoporous
Hydroxyapatites and Their Antimicrobial Properties. Colloids Surf., B.

[8] Petrakova N. V., Demina A. Y., Zobkova Y. O., Pechenkova N. S., Lysenkov A. S., Pavlov I. S., Chumakov R. G., Fedorov S. V., Egorov A. A., Kozyukhin S. A., Chizhevskaya S. V., Zhukov A. V., Fomina A. A., Filatova B. S. M., Darija G., Komlev V. S. (2025). Effect of Sodium and Cerium Co-Doping
on Microstructure
and Luminescent Properties of Hydroxyapatite Ceramics. J. Alloys Compd..

[9] Bazin T., Gaudon M., Champion E., Julien I., Prestipino C., Figueroa S. J., Duttine M., Demourgues A. (2024). Copper Versatility
in Hydroxyapatite: Valence States, Clusters, and Optical Absorption
Properties. Inorg. Chem..

[10] Moisa M., Balasea B. V., Imre M., Vitelaru C., Radulescu R., Duica F., Rus F., Pana I., Muscurel C., Popa A., Ripszky A., Cernega A., Vladescu A., Pituru S. (2025). Insights into the Biocompatibility
of Human Gingival
Fibroblasts Cultured on a Hydroxyapatite-Coated New Ti-Nb Alloy. New
Perspectives For Dentistry?. Ceram. Int..

[11] Rajan S. T., Das M., Arockiarajan A. (2022). Biocompatibility
and Corrosion Evaluation of Niobium
Oxide Coated AZ31B Alloy for Biodegradable Implants. Colloids Surf., B.

[12] Mestieri L. B., Gomes-Cornélio A. L., Rodrigues E. M., Faria G., Guerreiro-Tanomaru J.
M., Tanomaru-Filho M. (2017). Cytotoxicity
and Bioactivity of Calcium Silicate Cements Combined with Niobium
Oxide in Different Cell Lines. Braz. Dent. J..

[13] Bergschmidt P., Bader R., Finze S., Schulze C., Kundt G., Mittelmeier W. (2011). Comparative
Study of Clinical and Radiological Outcomes
of Unconstrained Bicondylar Total Knee Endoprostheses with Anti-Allergic
Coating. Open Orthop. J..

[14] Leitune V. C. B., Collares F. M., Takimi A., de Lima G. B., Petzhold C. L., Bergmann C. P., Samuel S. M. W. (2013). Niobium
Pentoxide as a Novel Filler
for Dental Adhesive Resin. J. Dent..

[15] Pradhan D., Wren A., Misture S., Mellott N. (2016). Investigating
the Structure
and Biocompatibility of Niobium and Titanium Oxides as Coatings for
Orthopedic Metallic Implants. Mater. Sci. Eng.,
C.

[16] Palaniappan S., Sharma S., Radhakrishnan M., Krishna K. M., Joshi S. S., Banerjee R., Dahotre N. B. (2025). Process
Thermokinetics Influenced
Microstructure and Corrosion Response in Additively In-Situ Manufactured
Ti-Nb-Sn and Ti-Nb Alloys. J. Manuf. Process..

[17] Tan J., Li J., Cao B., Wu J., Luo D., Ran Z., Deng L., Li X., Jiang W., Xie K., Wang L., Hao Y. (2022). Niobium Promotes
Fracture Healing
in Rats by Regulating the PI3K-Akt Signalling Pathway: An *In Vivo* and *In Vitro* Study. J. Orthop. Transl..

[18] Gostin P. F., Helth A., Voss A., Sueptitz R., Calin M., Eckert J., Gebert A. (2013). Surface Treatment,
Corrosion Behavior,
and Apatite-Forming Ability of Ti-45Nb Implant Alloy. J. Biomed. Mater. Res. B: Appl. Biomater..

[19] de
Souza Balbinot G., Leitune V. C. B., da Cunha Bahlis E. A., Ponzoni D., Visioli F., Collares F. M. (2023). Niobium-Containing
Bioactive Glasses Modulate Alkaline Phosphatase Activity During Bone
Repair. J. Biomed. Mater. Res. B: Appl. Biomater..

[20] Kushwaha M., Pan X., Holloway J. A., Denry I. L. (2012). Differentiation of Human Mesenchymal
Stem Cells on Niobium-Doped Fluorapatite Glass-Ceramics. Dent. Mater..

[21] Obata A., Takahashi Y., Miyajima T., Ueda K., Narushima T., Kasuga T. (2012). Effects of Niobium Ions Released
from Calcium Phosphate
Invert Glasses Containing Nb_2_O_5_ on Osteoblast-Like
Cell Functions. ACS Appl. Mater. Interfaces.

[22] de
Moraes E. G., Andrade K. L., Ribeiro L. F. B., da
Costa Laqua L. A., da Silva D. F., Arcaro S., Hiratsuka L. S., Tada D., Faita F. L., de Oliveira A. P. N., Machado R. A. F. (2025). Influence of Graphene, Hydroxyapatite,
and Hydroxyapatite-Niobium Pentoxide Nanoparticles on Polycaprolactone
Electrospun Fibers Mats Properties. J. Appl.
Polym. Sci..

[23] Safavi M. S., Khalil-Allafi J., Motallebzadeh A., Volpini C., Khalili V., Visai L. (2023). Encouraging
Tribomechanical and Biological Responses of Hydroxyapatite
Coatings Reinforced by Various Levels of Niobium Pentoxide Particles. Mater. Adv..

[24] Bonadio T. G. M., Sato F., Medina A., Weinand W., Baesso M., Lima W. (2013). Bioactivity and Structural Properties of Nanostructured Bulk Composites
Containing Nb_2_O_5_ and Natural Hydroxyapatite. J. Appl. Phys..

[25] Tamai M., Isama K., Nakaoka R., Tsuchiya T. (2007). Synthesis of a Novel *β*-Tricalcium
Phosphate/Hydroxyapatite Biphasic Calcium
Phosphate Containing Niobium Ions and Evaluation of its Osteogenic
Properties. J. Artif. Organs.

[26] Thiyagarajan P., Shanmugharaj A., Alagesan T., Padmapriya A., Kalaivani R. (2023). DFT Theoretical
and Experimental Studies Unraveling
the Structural and Electronic Properties of Niobium Doped Calcium
Apatite Ceramics. Mater. Today Commun..

[27] Capanema N. S., Mansur A. A., Carvalho S. M., Silva A. R., Ciminelli V. S., Mansur H. S. (2015). Niobium-Doped Hydroxyapatite
Bioceramics: Synthesis,
Characterization and In Vitro Cytocompatibility. Materials.

[28] Korzeniewski W., Witkowska A. (2023). Dissolution
of Nb-Doped Hydroxyapatite Prepared via
Low-Temperature Mechanochemical Method: Spectroscopy Studies. Nucl. Instrum. Methods Phys. Res., Sect. B.

[29] da
Silva O. G., Alves M. M., Dos Santos I. M. G., Fonseca M. G., Jaber M. (2017). Mesoporous Calcium Phosphate Using
Casein as a Template: Application to Bovine Serum Albumin Sorption. Colloids Surf., B.

[30] Ravel B., Newville M. (2005). ATHENA, ARTEMIS, HEPHAESTUS:
Data Analysis for X-ray
Absorption Spectroscopy Using IFEFFIT. J. Synchrotron
Radiat..

[31] Wayne, P. C. In Clinical and Laboratory Standards Institute (CLSI) Method for Dilution Antimicrobial Susceptibility Tests for Bacteria that Grow Aerobically; Approved Standard-8th ed, CLSI Document M07-A8, USA, 2009.

[32] Rodríguez-Melcón C., Alonso-Calleja C., García-Fernández C., Carballo J., Capita R. (2022). Minimum Inhibitory Concentration
(MIC) and Minimum Bactericidal Concentration (MBC) for Twelve Antimicrobials
(Biocides and Antibiotics) in Eight Strains of Listeria monocytogenes. Biology.

[33] Balouiri M., Sadiki M., Ibnsouda S. K. (2016). Methods for *In Vitro* Evaluating Antimicrobial Activity: A Review. J. Pharm. Anal..

[34] de
Amorim M. O., Júnior J. C. C.
S., Ruiz Y., Andrade J. (2021). Synthesis and Characterization of Niobium-Doped Fish
Scale-Derived Hydroxyapatite by Physical Ultrasound Interference. Cerâmica.

[35] Paduraru A. V., Oprea O., Musuc A. M., Vasile B. S., Iordache F., Andronescu E. (2021). Influence
of Terbium Ions and Their Concentration on
the Photoluminescence Properties of Hydroxyapatite for Biomedical
Applications. Nanomaterials.

[36] Shannon R. D. (1976). Revised
Effective Ionic Radii and Systematic Studies of Interatomic Distances
in Halides and Chalcogenides. Acta Crystallogr.
A.

[37] Terra J., Dourado E. R., Eon J.-G., Ellis D. E., Gonzalez G., Rossi A. M. (2009). The Structure of
Strontium-Doped Hydroxyapatite: An
Experimental and Theoretical Study. Phys. Chem.
Chem. Phys..

[38] Matos M., Terra J., Ellis D. E. (2010). Mechanism
of Zn Stabilization in
Hydroxyapatite and Hydrated (0 0 1) Surfaces of Hydroxyapatite. J. Phys.: Condens. Matter..

[39] Veselinović L., Karanović L., Stojanović Z., Bračko I., Marković S., Ignjatović N., Uskoković D. (2010). Crystal Structure
of Cobalt-Substituted Calcium Hydroxyapatite Nanopowders Prepared
by Hydrothermal Processing. J. Appl. Crystallogr..

[40] Matsunaga K. (2008). Theoretical
Investigation of the Defect Formation Mechanism Relevant to Nonstoichiometry
in Hydroxyapatite. Phys. Rev. B.

[41] Cacciotti, I. Cationic and Anionic Substitutions in Hydroxyapatite. In Handbook of Bioceramics and Biocomposites; Springer, 2014; pp 1–68 10.1007/978-3-319-09230-0_7-1.

[42] Ignjatović N.
L., Mančić L., Vuković M., Stojanović Z., Nikolić M. G., Škapin S., Jovanović S., Veselinović L., Uskoković V., Lazić S., Marković S., Lazarević M. M., Uskoković D. P. (2019). Rare-earth
(Gd^3+^, Yb^3+^/Tm^3+^, Eu^3+^) Co-Doped Hydroxyapatite as Magnetic, Up-Conversion and Down-Conversion
Materials for Multimodal Imaging. Sci. Rep..

[43] Oliveira C., de Oliveira A. L. M., Chantelle L., Cavalcanti G. R., Landers R., Medina-Carrasco S., Orta M. D. M., Silva
Filho E. C., Jaber M., Fonseca M. G. (2022). Functionalization
of the Hydroxyapatite Surface with ZnO for Alizarin Immobilization. Appl. Surf. Sci..

[44] Oliveira C., de Oliveira A. L. M., Chantelle L., Landers R., Medina-Carrasco S., Orta M. D. M., Silva Filho E. C., Fonseca M. G., Zinc I. I. (2021). Zinc (II)
Modified Hydroxyapatites for Tetracycline Removal: Zn (II) Doping
or ZnO Deposition and Their Influence in the Adsorption. Polyhedron.

[45] Pereira M., França D., Araújo R. C., Silva Filho E. C., Rigaud B., Fonseca M., Jaber M. (2020). Amino Hydroxyapatite/Chitosan
Hybrids Reticulated with Glutaraldehyde at Different pH Values and
Their Use for Diclofenac Removal. Carbohydr.
Polym..

[46] Beasley M. M., Bartelink E. J., Taylor L., Miller R. M. (2014). Comparison of Transmission
FTIR, ATR, and DRIFT Spectra: Implications for Assessment of Bone
Bioapatite Diagenesis. J. Archaeol. Sci..

[47] Gomes S., Nedelec J.-M., Jallot E., Sheptyakov D., Renaudin G. (2011). Unexpected Mechanism of Zn^2+^ Insertion in
Calcium Phosphate Bioceramics. Chem. Mater..

[48] Manatunga D. C., de Silva R. M., de Silva K. M. N., de Silva N., Premalal E. (2018). Metal and
Polymer-Mediated Synthesis of Porous Crystalline Hydroxyapatite Nanocomposites
for Environmental Remediation. R. Soc. Open
Sci..

[49] Mercado D. F., Magnacca G., Malandrino M., Rubert A., Montoneri E., Celi L., Bianco Prevot A., Gonzalez M. C. (2014). Paramagnetic Iron-Doped
Hydroxyapatite Nanoparticles with Improved Metal Sorption Properties.
A Bioorganic Substrates-Mediated Synthesis. ACS Appl. Mater. Interfaces.

[50] Madupalli H., Pavan B., Tecklenburg M. M. (2017). Carbonate Substitution in the Mineral
Component of Bone: Discriminating the Structural Changes, Simultaneously
Imposed by Carbonate in A and B Sites of Apatite. J. Solid State Chem..

[51] Santos R. D. S., dos S Rezende M. V. (2014). Atomistic Simulation of Intrinsic
Defects and Trivalent and Tetravalent Ion Doping in Hydroxyapatite. Condens. Matter Phys..

[52] Riaz M., Zia R., Ijaz A., Hussain T., Mohsin M., Malik A. (2018). Synthesis
of Monophasic Ag Doped Hydroxyapatite and Evaluation of Antibacterial
Activity. Mater. Sci. Eng., C.

[53] Silva C., Sombra A. (2004). Raman Spectroscopy
Measurements of Hydroxyapatite Obtained
by Mechanical Alloying. J. Phys. Chem. Solids.

[54] Dal
Sasso G., Angelini I., Maritan L., Artioli G. (2018). Raman Hyperspectral
Imaging as an Effective and Highly Informative Tool to Study the Diagenetic
Alteration of Fossil Bones. Talanta.

[55] Chen W., Yu Z., Pang J., Yu P., Tan G., Ning C. (2017). Fabrication
of Biocompatible Potassium Sodium Niobate Piezoelectric Ceramic as
an Electroactive Implant. Materials.

[56] Zhu H., Zheng Z., Gao X., Huang Y., Yan Z., Zou J., Yin H., Zou Q., Kable S. H., Zhao J., Xi Y., Martens W. N., Frost R. L. (2006). Structural Evolution in a Hydrothermal
Reaction Between Nb_2_O_5_ and NaOH Solution: From
Nb_2_O_5_ Grains to Microporous Na_2_Nb_2_O_6_·2/3H_2_O Fibers and NaNbO_3_ Cubes. J. Am. Chem. Soc..

[57] Carniti P., Gervasini A., Tiozzo C., Guidotti M. (2014). Niobium-Containing
Hydroxyapatites as Amphoteric Catalysts: Synthesis, Properties, and
Activity. ACS Catal..

[58] Scotti N., Ravasio N., Evangelisti C., Psaro R., Penso M., Niphadkar P. S., Bokade V. V., Guidotti M. (2019). Epoxidation of Karanja
(Millettia pinnata) Oil Methyl Esters in the Presence of Hydrogen
Peroxide Over a Simple Niobium-Containing Catalyst. Catalysts.

[59] Wu J.-F., Ramanathan A., Kersting R., Jystad A., Zhu H., Hu Y., Marshall C. P., Caricato M., Subramaniam B. (2020). Enhanced Olefin
Metathesis Performance of Tungsten and Niobium Incorporated Bimetallic
Silicates: Evidence of Synergistic Effects. ChemCatChem.

[60] Bharath G., Latha B. S., Alsharaeh E. H., Prakash P., Ponpandian N. (2017). Enhanced Hydroxyapatite
Nanorods Formation on Graphene Oxide Nanocomposite as a Potential
Candidate for Protein Adsorption, pH Controlled Release and an Effective
drug delivery platform for cancer therapy. Anal.
Methods.

[61] Lowry N., Han Y., Meenan B., Boyd A. (2017). Strontium and Zinc Co-Substituted
Nanophase Hydroxyapatite. Ceram. Int..

[62] Xie L., Yang Y., Fu Z., Li Y., Shi J., Ma D., Liu S., Luo D. (2019). Fe/Zn-Modified
Tricalcium Phosphate
(TCP) Biomaterials: Preparation and Biological Properties. RSC Adv..

[63] Xue S., Deng H., Xie Q., Hu Y., Yan J., Zhao X., Wang F., Zhang Q., Luo L., Deng C., He C., Lin D., Li S., Wang X., Luo H. (2019). Giant Tunability of Upconversion
Photoluminescence inEr^3+^-Doped (K,Na)­NbO_3_ Single
Crystals. Nanoscale.

[64] Atuchin V., Kalabin I., Kesler V., Pervukhina N. (2005). Nb 3d and
O 1s Core Levels and Chemical Bonding in Niobates. J. Electron Spectrosc. Relat. Phenom..

[65] Guo J., Ren J., Cheng R., Dong Q., Gao C., Zhang X., Guo S. (2018). Growth, Structural
and Thermophysical Properties of TbNbO_4_ Crystals. CrystEngComm.

[66] Lee A. Y., Powell C. J., Gorham J. M., Morey A., Scott J. H. J., Hanisch R. J. (2024). Development of the NIST X-ray Photoelectron Spectroscopy
(XPS) Database, Version 5. Data Sci. J..

[67] Tofoni A., Tavani F., Persson I., D’Angelo P. (2023). P K-edge XANES
Calculations of Mineral Standards: Exploring the Potential of Theoretical
Methods in the Analysis of Phosphorus Speciation. Inorg. Chem..

[68] Hilger D. M., Hamilton J. G., Peak D. (2020). The Influences of Magnesium Upon
Calcium Phosphate Mineral Formation and Structure as Monitored by
X-ray and Vibrational Spectroscopy. Soil Syst..

[69] Liu J., Yang J., Cade-Menun B. J., Hu Y., Li J., Peng C., Ma Y. (2017). Molecular Speciation
and Transformation
of Soil Legacy Phosphorus with and Without Long-Term Phosphorus Fertilization:
Insights From Bulk and Microprobe Spectroscopy. Sci. Rep..

[70] Persson I., Klysubun W., Lundberg D. (2019). A K-edge P
XANES Study of Phosphorus
Compounds in Solution. J. Mol. Struct..

[71] Bollaert Q., Chassé M., Elnaggar H., Juhin A., Courtin A., Galoisy L., Quantin C., Retegan M., Vantelon D., Calas G. (2023). Niobium Speciation
in Minerals Revealed by L_2,3_-Edges
XANES Spectroscopy. Am. Mineral..

[72] Wagner, C. D. The NIST X-ray Photoelectron Spectroscopy (XPS) Database, NIST Technical Note Number 1289; US Department of Commerce: Gaithersburg, MD, 1991.

[73] Kubouchi Y., Hayakawa S., Namatame H., Hirokawa T. (2012). Direct Observation
of Fractional Change of Niobium Ionic Species in a Solution by Means
of X-ray Absorption Fine Structure Spectroscopy. X-Ray Spectrom..

[74] Wang B., Zhao Y., Banis M. N., Sun Q., Adair K. R., Li R., Sham T.-K., Sun X. (2018). Atomic Layer Deposition of Lithium
Niobium Oxides as Potential Solid-State Electrolytes for Lithium-Ion
Batteries. ACS Appl. Mater. Interfaces.

[75] Chantelle L., Kennedy B. J., de Oliveira C. P., Gouttefangeas F., Siu-Li M., Landers R., Ciorita A., Rostas A. M., dos Santos I. M. G., de Oliveira A. L. M. (2023). Europium
Induced Point Defects in
SrSnO_3_-Based Perovskites Employed as Antibacterial Agents. J. Alloys Compd..

[76] do
Nascimento J. L. A., Rostas A. M., Silva A., Kennedy B. J., Barbu-Tudoran L., Bocirnea A.-E., Santos I. M. G. d., Alves M. C. F., Menezes de Oliveira A.
L. (2025). Tailoring Structural
Distortions and Ionic Defects as Alternative Strategy to Modulate
Reactive Oxygen Species and Photocatalytic Activity in SnO_2_ Nanoparticles. Chem. Mater..

[77] Yin Z., Wu Y., Shi X., Xu Y. (2026). Nanomaterial-Mediated Antimicrobial
Mechanism of ROS and Its Application in Infectious Diseases. Chem. Eng. J..

[78] Wang M., Li M., Wang Y., Shao Y., Zhu Y., Yang S. (2021). Efficient
Antibacterial Activity of Hydroxyapatite Through ROS Generation Motivated
by Trace Mn­(III) Coupled H Vacancies. J. Mater.
Chem. B.

[79] Saranya S., Rani M. P. (2021). Sol Gel Synthesis
of Niobium Influence on Hydroxyapatite:
A View of In Vitro, Structural, Morphological and Studies for Biomedical
Applications. Mater. Today Proc..

