# Supplementary material for: Effect of Nb5+ Doping on Structural, Electronic, and Antibacterial Properties of Hydroxyapatite
Source: Inorg Chem. 2026 May 25;65(22):12371–86. doi: 10.1021/acs.inorgchem.6c01213 (PMC13250989; doi:10.1021/acs.inorgchem.6c01213)
Supplement: Supplementary file 1 [file ic6c01213_si_001.pdf]

Supporting Information:

Effect of Nb^{5+} Doping on Structural, Electronic, and Antibacterial Properties of Hydroxyapatite

Rafael Araújo,[†] André Luiz Menezes de Oliveira,[*,‡,¶] Brendan James Kennedy,[§] Ary da Silva Maia,[‡] Lúcio Castellano,[‖] Fábio Correia Sampaio,[⊥] Santiago Medina-Carrasco,[#] María del Mar Orta Cuevas,[@] and Maria Gardênnia Fonseca[*,‡]

†*Núcleo de Pesquisa e Extensão LACOM, Dept. de Química, Universidade Federal da Paraíba, 58051-085, João Pessoa-PB, Brazil.*

‡*Núcleo de Pesquisa e Extensão LACOM, Dept. de Química, Universidade Federal da Paraíba, 58051-085, João Pessoa-PB, Brazil.*

¶*Laboratório Institucional de Microscopia Eletrônica e Caracterização de Materiais (LIME), Dept. de Engenharia de Materiais, Universidade Federal do Rio Grande do Norte, 58078-970, Natal-RN, Brazil.*

§*School of Chemistry, The University of Sydney, Sydney, NSW 2006, Australia.*

‖*Escola Técnica de Saúde, Universidade Federal da Paraíba, 58051-900, João Pessoa-PB, Brazil.*

⊥*Dept. de Clínica e Odontologia Social, Universidade Federal da Paraíba, 58051-900, João Pessoa-PB, Brazil.*

#*CITIUS Laboratorio de Rayos X, Universidad de Sevilla, 4B 41012, Seville, Andalucía, Spain.*

@*Facultad de Farmacia, Dpt.de Química Análitica, Universidad de Sevilla, E 41012, Seville, Andalucía, Spain.*

E-mail: andrel_ltm@hotmail.com; mgardennia@quimica.ufpb.br

Table S1: Refined atomic positions and Ca/Nb occupancies for HapNbx (x = 0, 1, 3, 5, 7, and 10 mol%) samples. The atomic displacement parameters for the four oxygen sites were constrained to be equal. The hydrogen atoms were not considered in the refinements due to their inconsequential impact on the data.

Atom	x	y	z	Occup	Uiso (Å^2)
			**Undoped Hap**		
Ca1	0.3333	0.6667	-0.0018(8)	1.0	0.026(14)
Ca2	0.2465(5)	0.9913(7)	0.25	1.0	0.034(10)
P1	0.3973(6)	0.3693(6)	0.25	1.0	0.036(16)
O1	0.3274(11)	0.4782(11)	0.25	1.0	0.033(2)
O2	0.5862(12)	0.4651(13)	0.25	1.0	0.033(2)
O3	0.3395(8)	0.2545(10)	0.0707(8)	1.0	0.033(2)
O4	0.0	0.0	0.1974(27)	0.5	0.033(2)
			**HapNb1%**		
Ca1	0.3333	0.6667	0.0015(9)	0.974(14)	0.023(2)
Ca2	0.2452(5)	0.9921(8)	0.25	0.959(7)	0.040(2)
Nb2	0.2452(5)	0.9921(8)	0.25	0.041(7)	0.040(2)
P1	0.3970(8)	0.3702(7)	0.25	1.0	0.033(2)
O1	0.3277(12)	0.4816(14)	0.25	1.0	0.031(2)
O2	0.5855(14)	0.4640(15)	0.25	1.0	0.031(2)
O3	0.3406(11)	0.2568(11)	0.0725(10)	1.0	0.031(2)
O4	0.0	0.0	0.2009(30)	0.5	0.031(2)
			**HapNb3%**		
Ca1	0.3333	0.6667	-0.0016(11)	0.977(18)	0.024(3)
Ca2	0.2440(6)	0.9894(10)	0.25	0.944(9)	0.042(2)
Nb2	0.2440(6)	0.9894(10)	0.25	0.056(9)	0.042(2)
P1	0.3985(9)	0.3706(9)	0.25	1.0	0.034(3)
O1	0.3271(15)	0.4810(17)	0.25	1.0	0.032(3)
O2	0.5864(17)	0.4648(19)	0.25	1.0	0.032(3)
O3	0.3401(14)	0.2569(13)	0.0725(11)	1.0	0.032(3)
O4	0.0	0.0	0.201(4)	0.5	0.032(3)
			**HapNb5%**		
Ca1	0.3333	0.6667	0.0022(12)	0.992(20)	0.024(3)
Ca2	0.2440(7)	0.9903(10)	0.25	0.925(9)	0.043(2)
Nb2	0.2440(7)	0.9903(10)	0.25	0.075(9)	0.043(2)
P1	0.3984(10)	0.3714(9)	0.25	1.0	0.027(3)
O1	0.3328(19)	0.4793(19)	0.25	1.0	0.027(3)
O2	0.5824(19)	0.4633(21)	0.25	1.0	0.027(3)
O3	0.3377(15)	0.2569(14)	0.0771(13)	1.0	0.027(3)
O4	0.0	0.0	0.194(4)	0.5	0.027(3)

Table S2: ***Continuation*** **- Table S1**

Atom	x	y	z	Occup	Uiso (Å^2)
			**HapNb7%**		
Ca1	0.3333	0.6667	0.0039(16)	1.011(24)	0.023(4)
Ca2	0.2439(8)	0.9942(12)	0.25	0.877(12)	0.043(2)
Nb2	0.2439(8)	0.9942(12)	0.25	0.123(12)	0.043(2)
P1	0.3996(13)	0.3725(12)	0.25	1.0	0.023(3)
O1	0.3395(20)	0.4835(24)	0.25	1.0	0.020(4)
O2	0.5796(25)	0.4613(27)	0.25	1.0	0.020(4)
O3	0.3371(19)	0.2582(18)	0.0787(16)	1.0	0.020(4)
O4	0.0	0.0	0.198(5)	0.5	0.020(4)
			**HapNb10%**		
Ca1	0.3333	0.6667	0.0052(17)	1.034(18)	0.023(4)
Ca2	0.2422(8)	0.9951(13)	0.25	0.928(11)	0.037(2)
Nb2	0.2422(8)	0.9951(13)	0.25	0.072(11)	0.037(2)
P1	0.3859(13)	0.3680(14)	0.25	1.0	0.030(4)
O1	0.3565(28)	0.4957(23)	0.25	1.0	0.012(4)
O2	0.5764(22)	0.4532(22)	0.25	1.0	0.012(4)
O3	0.3449(14)	0.2611(16)	0.0811(16)	1.0	0.012(4)
O4	0.0	0.0	0.220(7)	0.5	0.012(4)

Table S3: Selected bond distances (Å) and bond angles (°) in the HapNb*x* (*x*= 0, 1, 3, 5, 7, and 10 mol%) samples.

	**Hap**	**HapNb1%**	**HapNb3%**	**HapNb5%**	**HapNb7%**	**HapNb10%**
Bond distance (Å)						
P-O(1)	1.474(12)	1.489(15)	1.495(19)	1.43(2)	1.42(3)	1.37(3)
P-O(2)	1.546(9)	1.535(7)	1.534(13)	1.504(15)	1.47(2)	1.461(13)
P-O(3) x 2	1.553(7)	1.539(10)	1.537(9)	1.516(10)	1.508(12)	1.562(18)
Ca(1)-O(1) x 3	2.467(8)	2.427(10)	2.444(13)	2.458(14)	2.446(18)	2.419(17)
Ca(1)-O(2) x 3	2.450(7)	2.460(8)	2.446(10)	2.478(11)	2.490(14)	2.463(15)
Ca(1)-O(3) x 3	2.834(7)	2.831(10)	2.832(12)	2.867(13)	2.884(17)	2.826(12)
Ca(2)-O(1)	2.675(12)	2.690(13)	2.663(17)	2.711(17)	2.80(2)	2.96(2)
Ca(2)-O(2)	2.363(10)	2.380(12)	2.380(15)	2.399(17)	2.42(2)	2.503(18)
Ca(2)-O(3) x 2	2.338(6)	2.355(6)	2.348(8)	2.374(8)	2.394(10)	2.433(10)
Ca(2)-O(3) x 2	2.510(9)	2.508(3)	2.528(12)	2.513(13)	2.490(15)	2.485(15)
Ca(2)-O(4) x 2	2.399(4)	2.374(4)	2.376(5)	2.382(6)	2.360(7)	2.321(7)
Bond angle (°)						
O(1)-P-O(2)	112.3(7)	112.5(7)	112.8(9)	112.0(10)	110.7(13)	120.7(10)
O(1)-P-O(3)	111.3(5)	111.7(5)	110.9(7)	111.3(8)	111.8(10)	105.6(15)
O(2)-P-O(3)	108.0(5)	107.4(6)	108.1(8)	109.1(9)	109.6(11)	103.7(14)
O(3)-P-O(3)	105.6(7)	105.8(8)	105.6(10)	103.7(11)	103.2(14)	101.2(10)

Table S4: FWHM and Crystallite diameter (*Dc*) values obtained by refining the XRD patterns using Le Bail method.

Samples	**Hap**	**HapNb1%**	**HapNb3%**	**HapNb5%**	**HapNb7%**	**HapNb10%**	
Phases	Hap	Hap	Hap	Hap	Hap	Hap	$CaNb_2O_6$
FWHM	0.7053	0.8166	0.9101	1.0261	1.1244	1.2461	
Dc	17.5(2)	14.88(18)	11.87(13)	11.34(13)	7.74(9)	10.92(14)	28.2(9)
GOF	1.16	1.06	1.11	1.09	1.08	1.14	
R_{wp}	11.25	10.10	10.18	9.69	9.13	9.57	
R_{Bragg}	0.764	0.605	0.653	0.596	0.581	0.599	0.714

Table S5: FWHM values of the IR absorption band at 1033 cm^{-1} for pristine Hap and HapNbx samples

**Sample**	**FWHM** (cm^{-1})
Hap	78.65
HapNb1%	83.84
HapNb3%	84.05
HapNb5%	85.84
HapNb7%	88.40
HapNb10%	89.65

Table S6: FWHM values of the Raman mode at 961 cm^{-1} for pristine Hap and HapNbx samples.

**Sample**	**FWHM** (cm^{-1})
Hap	7.96
HapNb1%	7.86
HapNb3%	7.46
HapNb5%	8.04
HapNb7%	10.60
HapNb10%	11.42

Table S7: Binding energy (BE) and spin-orbit coupling (ΔE) energy obtained by XPS analysis. BE and ΔE values are expressed in eV

	**Hap**	**HapNb1%**	**HapNb3%**	**HapNb5%**	**HapNb7%**	**HapNb10%**
Ca 2p3/2	347.27	34.37	347.32	347.34	347.35	347.25
Ca 2p1/2	350.82	350.88	350.77	350.83	350.84	350.76
ΔE 3.55	3.51	3.45	3.49	3.49	3.51	
P 2p3/2	133.05	133.02	133.58	133.24	133.06	132.95
P 2p1/2	134.04	134.05	135.01	134.45	134.15	134.03
ΔE	0.99	1.03	1.44	1.21	1.09	1.08
Nb 3d5/2	-	207.27	207.22	207.24	207.20	207.16
Nb 3d3/2	-	210.07	209.87	209.95	209.96	209.86
ΔE	-	2.80	2.65	2.71	2.76	2.70

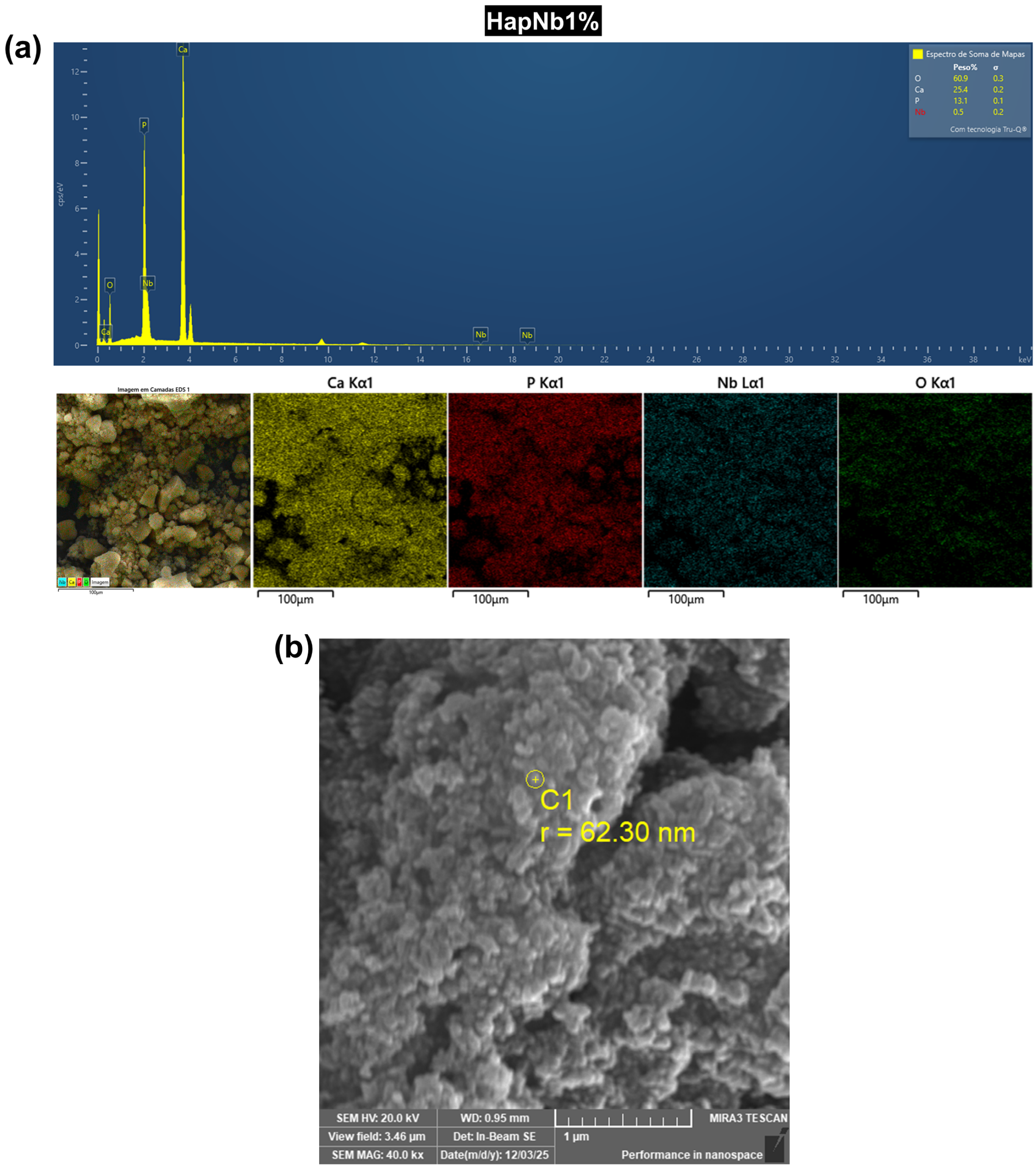

Figure S1: EDS spectra and elemental mapping (a) and FE-SEM image highlighting particle size (b) for sample HapNb1%.

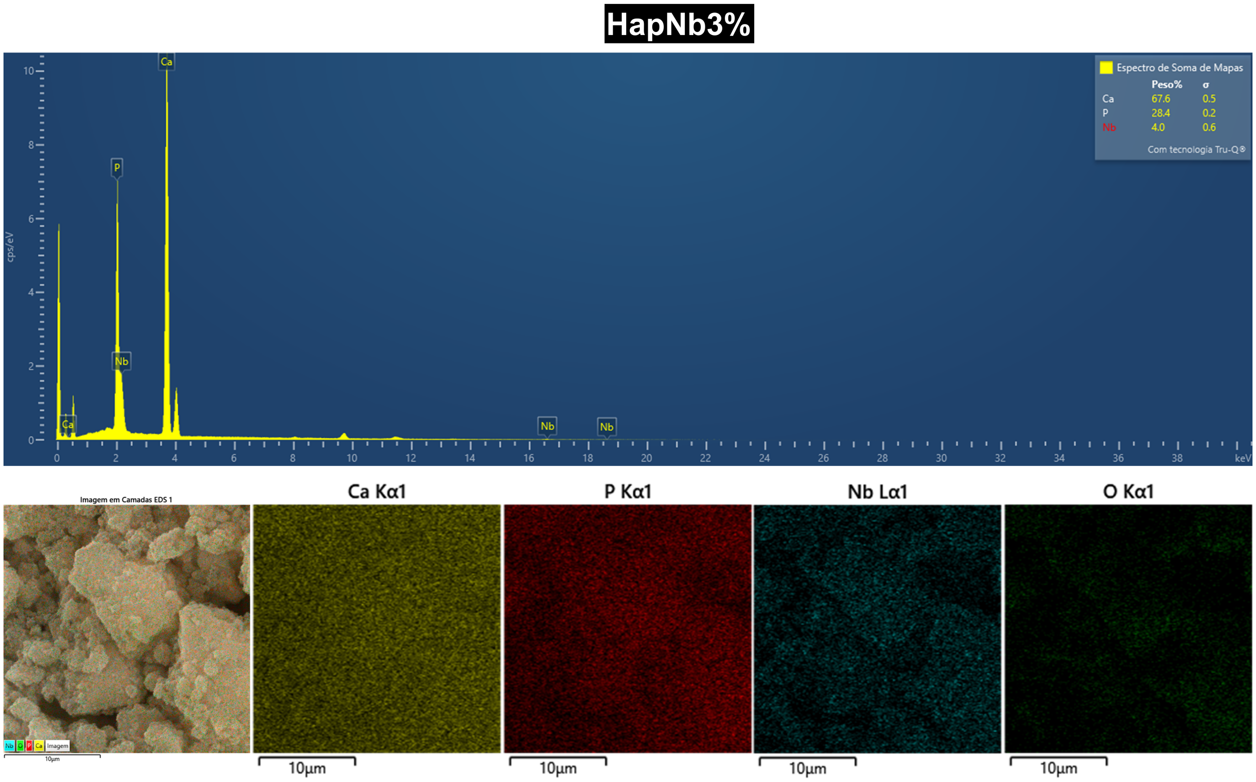

Figure S2: EDS spectra and elemental mapping for the sample HapNb3%.

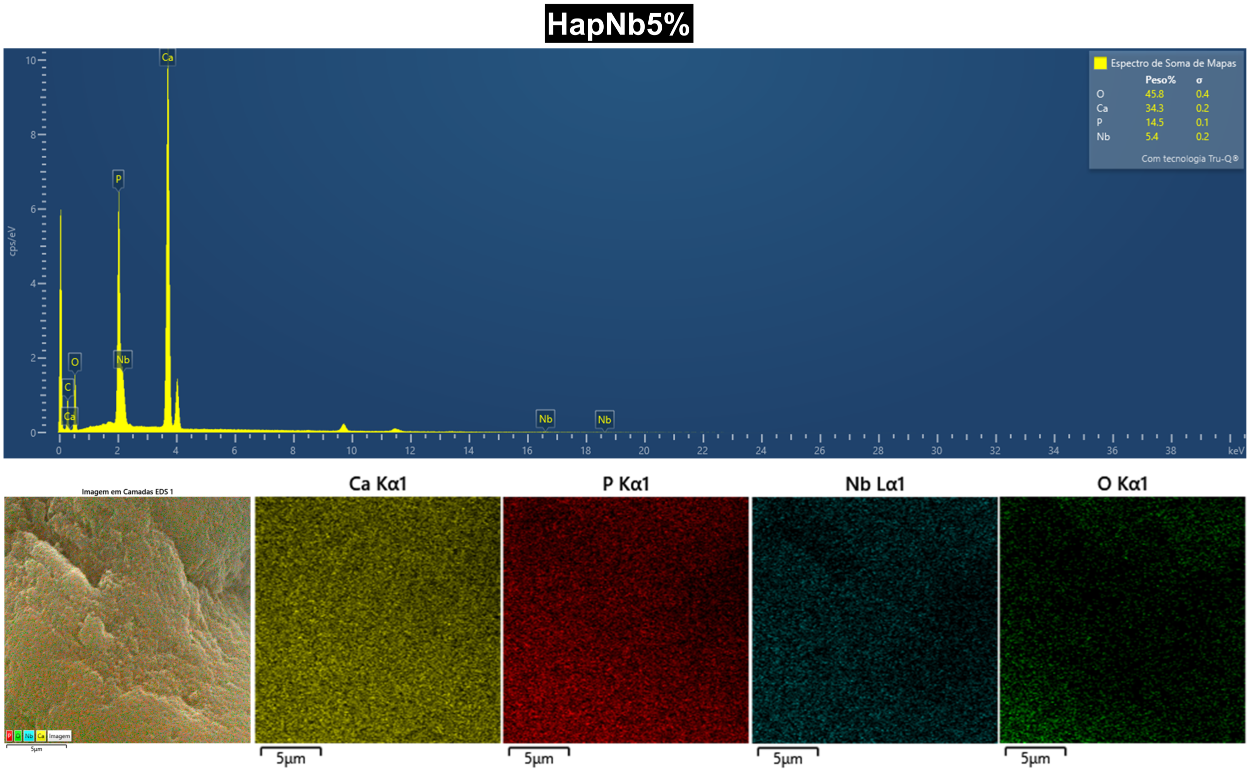

Figure S3: EDS spectra and elemental mapping for the sample HapNb5%.

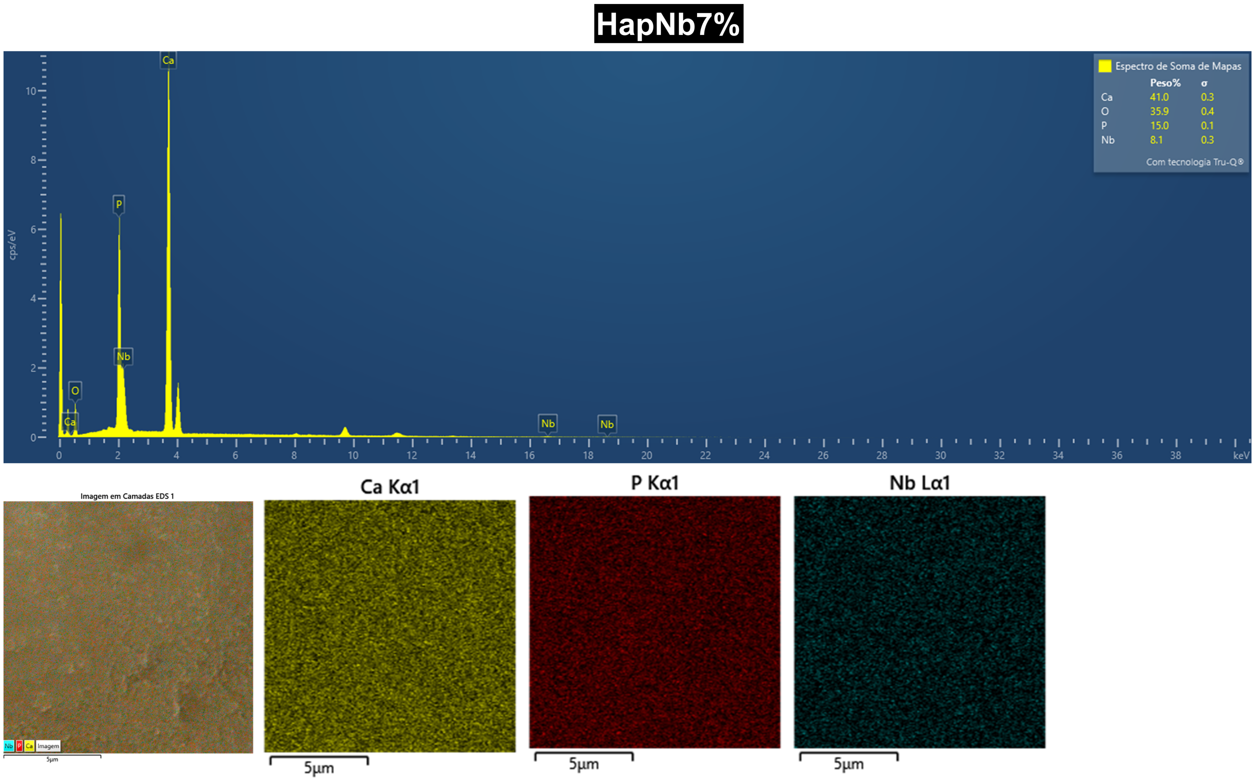

Figure S4: EDS spectra and elemental mapping for the sample HapNb7%.

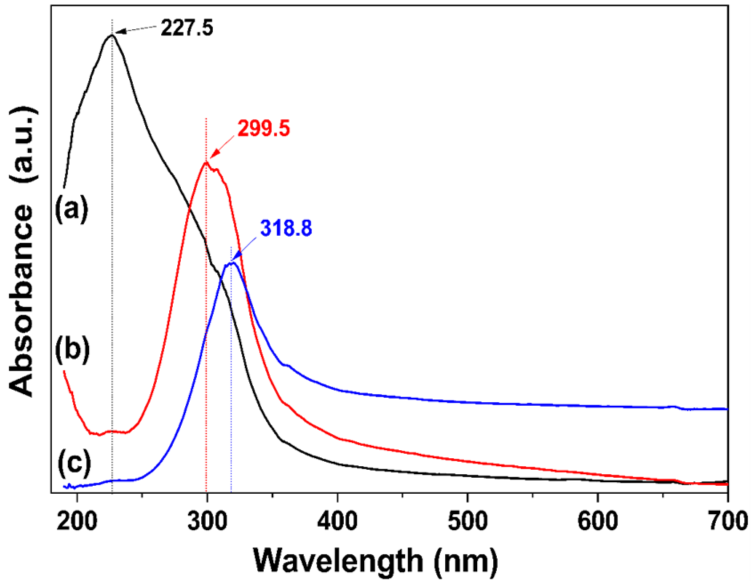

Figure S5: UV-vis spectra of (a) Hap, (b) HapNb5%, and (c) HapNb10% samples.

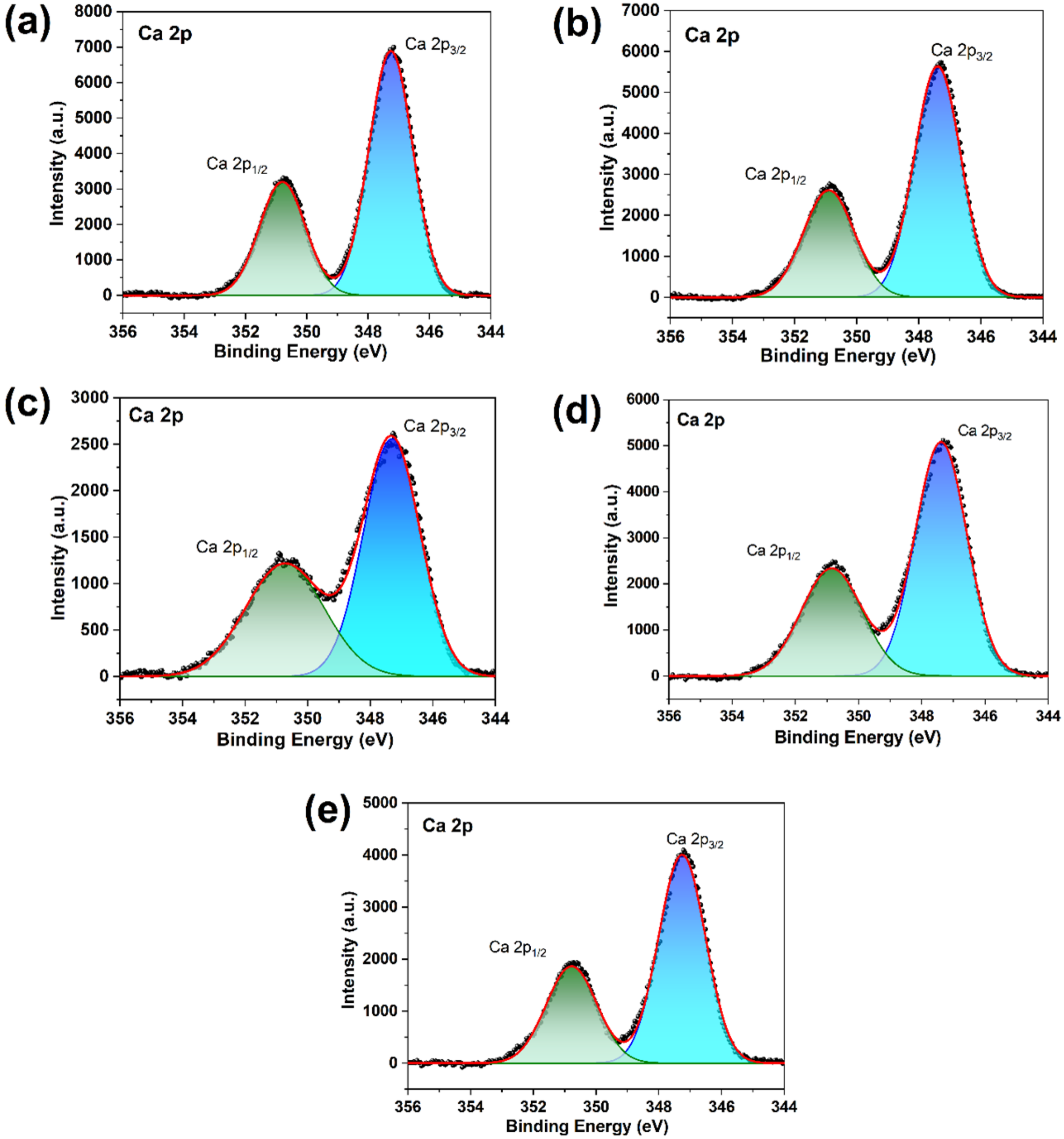

Figure S6: High-resolution XPS spectra deconvolution in the Ca 2p emission line for Hap (a), HapNb1% (b), HapNb3% (c), HapNb5% (d), and HapNb10% (e) samples.

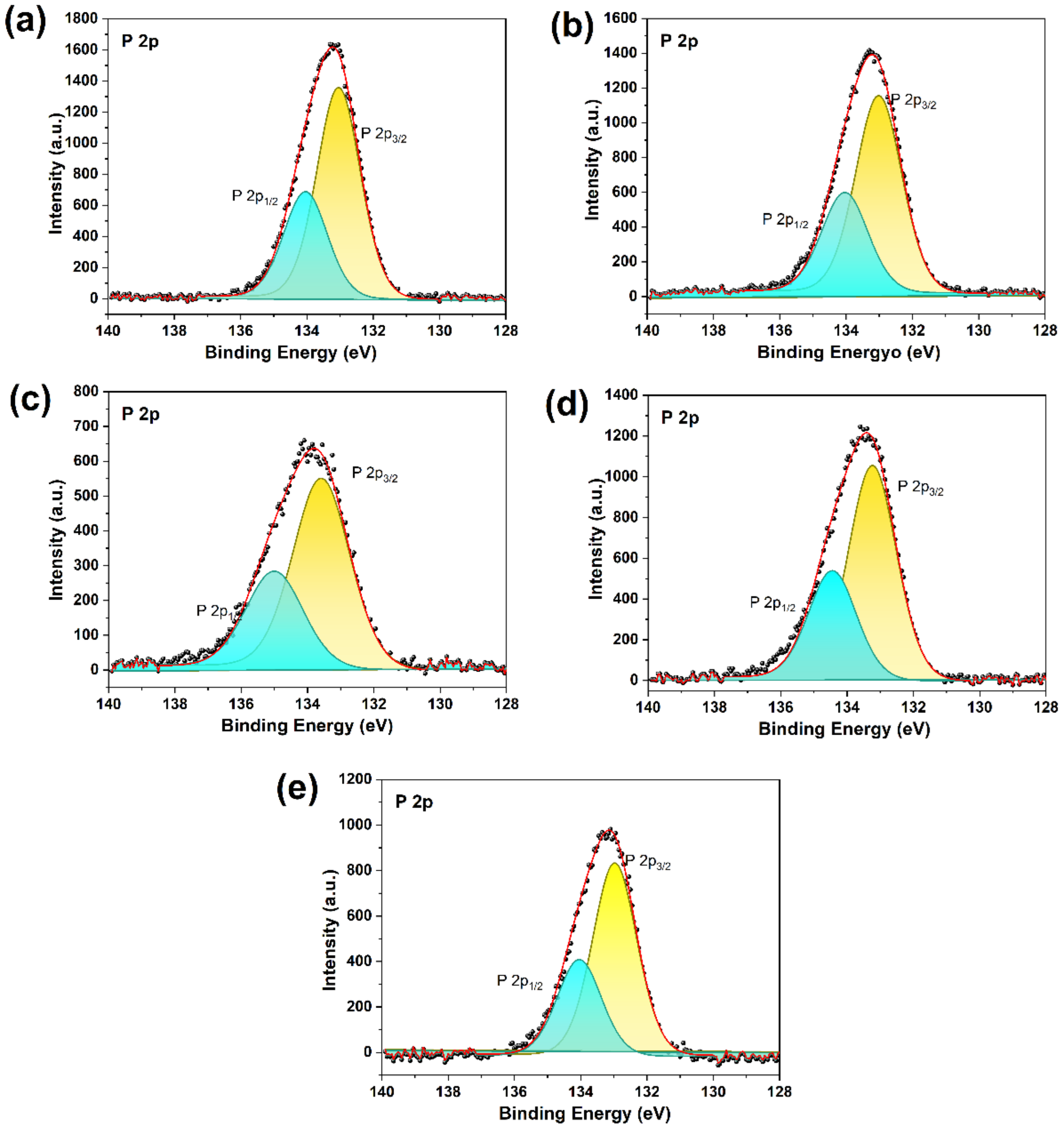

Figure S7: High-resolution XPS spectra deconvolution in P 2p emission line for Hap (a), HapNb1% (b), HapNb3% (c), HaNb5% (d), and HapNb10% (e) samples.

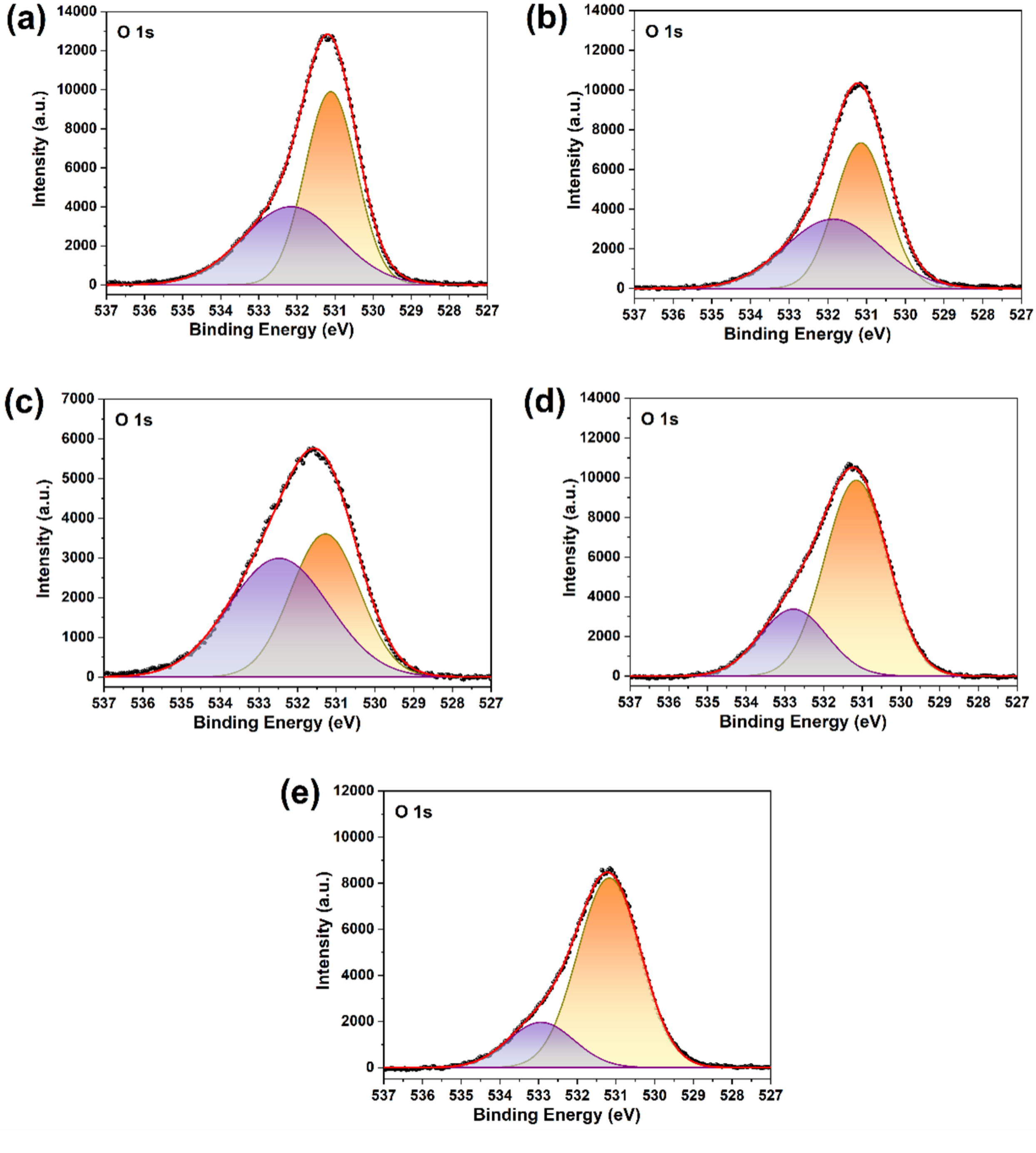

Figure S8: High-resolution XPS spectra deconvolution in O 1s emission line for Hap (a), HapNb1% (b), HapNb3% (c), HaNb5 (d), and HapNb10% (e) samples.

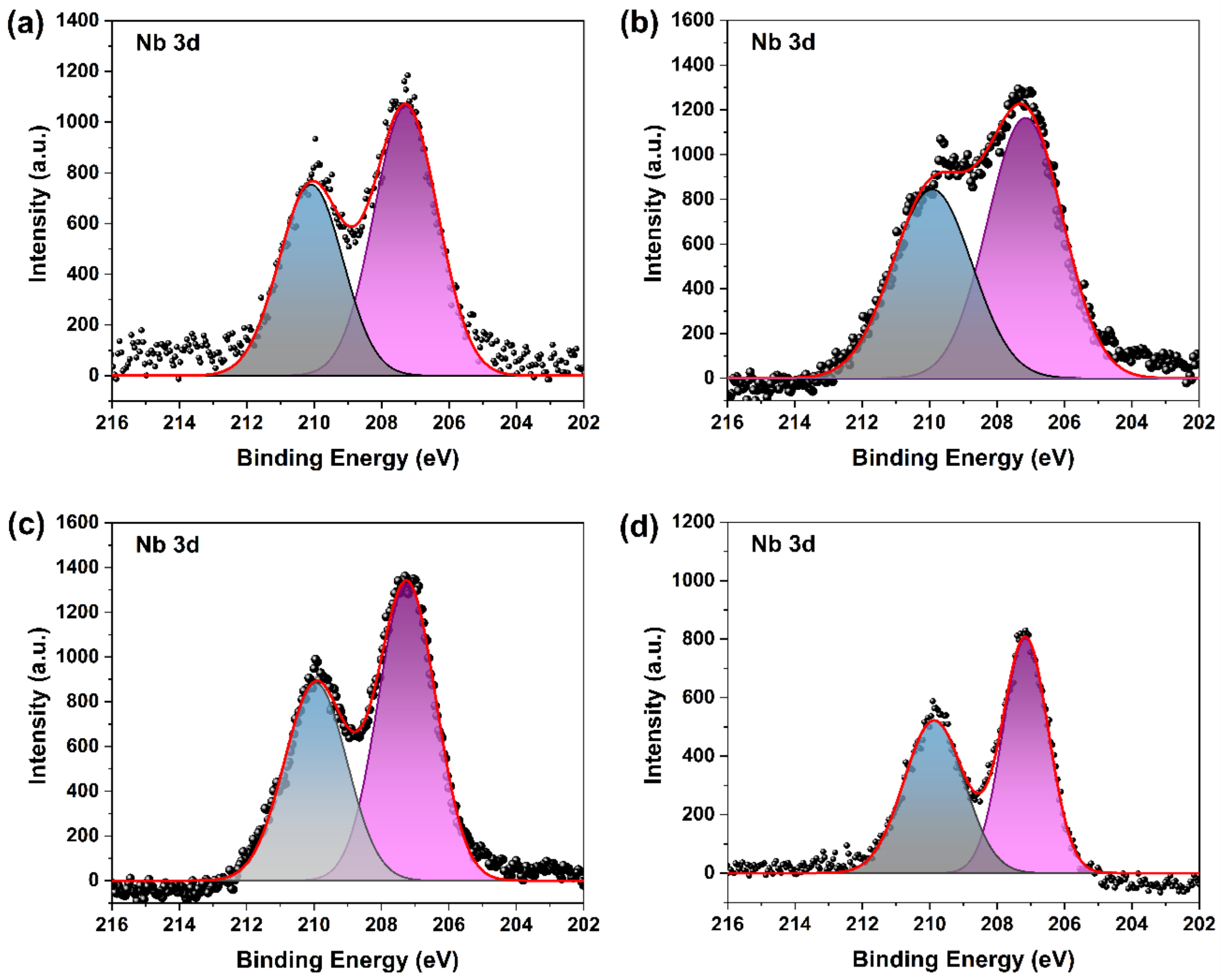

Figure S9: High-resolution XPS spectra deconvolution in Nb 3d emission line for HapNb1% (a), HapNb3% (b), HapNb5% (c), and HapNb10% (d) samples.

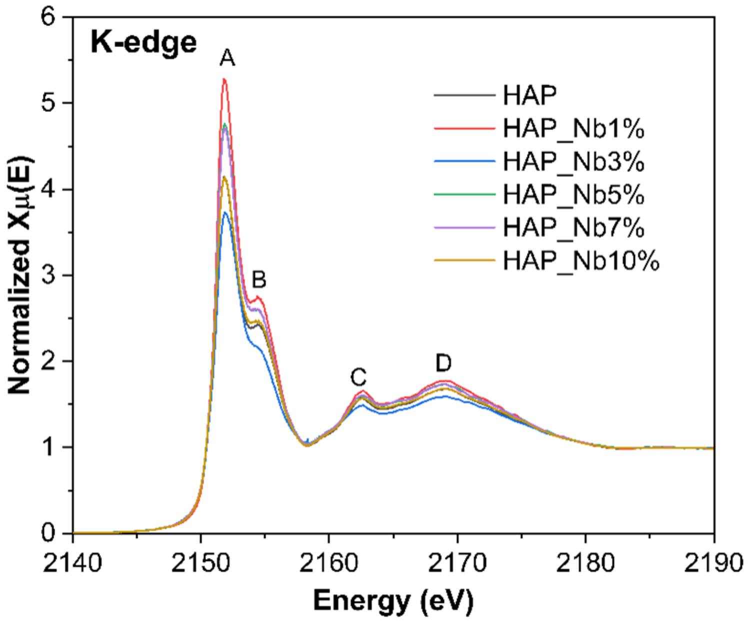

Figure S10: (Normalized Phosphorus K-edge XANES spectra from the pristine Hap and HapNbx samples.

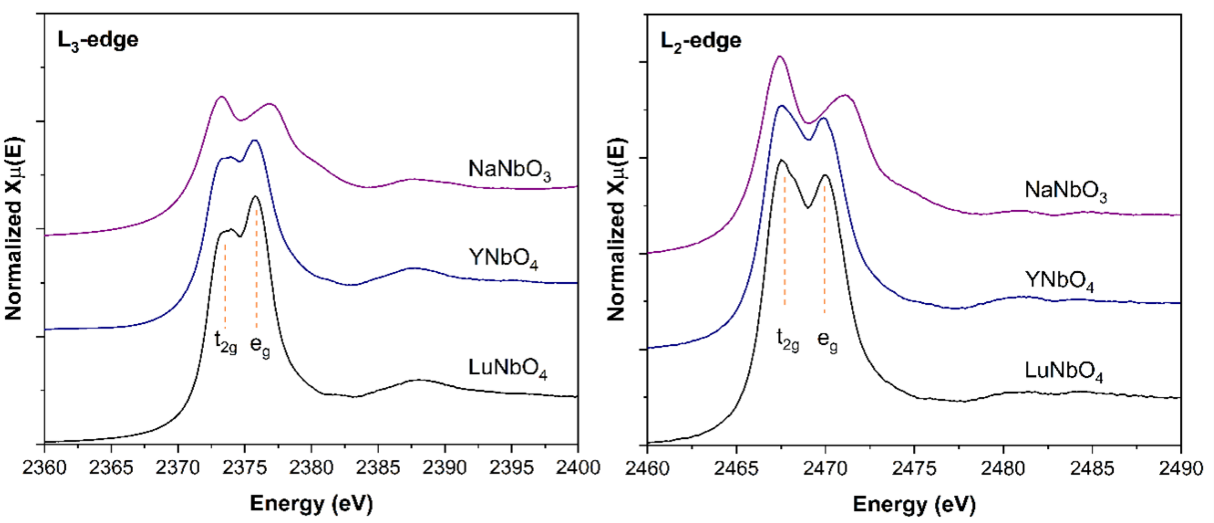

Figure S11: Normalized Niobium $L_{3,2}$-edges XANES spectra from the $LuNbO_4$ and $YNbO_4$ references for Nb^{5+} atoms in tetrahedral geometry and the $NaNbO_3$ reference for Nb^{5+} in octahedral geometry.
